# Flow pattern–dependent mitochondrial dynamics regulates the metabolic profile and inflammatory state of endothelial cells

**DOI:** 10.1172/jci.insight.159286

**Published:** 2022-09-22

**Authors:** Soon-Gook Hong, Junchul Shin, Soo Young Choi, Jeffery C. Powers, Benjamin M. Meister, Jacqueline Sayoc, Jun Seok Son, Ryan Tierney, Fabio A. Recchia, Michael D. Brown, Xiaofeng Yang, Joon-Young Park

**Affiliations:** 1 Cardiovascular Research Center, Lewis Katz School of Medicine, and; 2 Department of Kinesiology, College of Public Health, Temple University, Philadelphia, Pennsylvania, USA.; 3 Laboratory of Perinatal Kinesioepigenetics, Department of Obstetrics, Gynecology and Reproductive Sciences, University of Maryland School of Medicine, Baltimore, Maryland, USA.; 4 Institute of Clinical Physiology, National Research Council, Pisa, Italy.; 5 Department of Kinesiology, School of Public Health, University of Maryland, College Park, Maryland, USA.; 6 Robbins College of Health and Human Sciences, Baylor University, Waco, Texas, USA.; 7 Institute of Life Sciences, Scuola Superiore Sant’Anna, Pisa, Italy.

**Keywords:** Vascular Biology, Atherosclerosis, Endothelial cells, Mitochondria

## Abstract

Endothelial mitochondria play a pivotal role in maintaining endothelial cell (EC) homeostasis through constantly altering their size, shape, and intracellular localization. Studies show that the disruption of the basal mitochondrial network in EC, forming excess fragmented mitochondria, implicates cardiovascular disease. However, cellular consequences underlying the morphological changes in the endothelial mitochondria under distinctively different, but physiologically occurring, flow patterns (i.e., unidirectional flow [UF] versus disturbed flow [DF]) are largely unknown. The purpose of this study was to investigate the effect of different flow patterns on mitochondrial morphology and its implications in EC phenotypes. We show that mitochondrial fragmentation is increased at DF-exposed vessel regions, where elongated mitochondria are predominant in the endothelium of UF-exposed regions. DF increased dynamin-related protein 1 (Drp1), mitochondrial reactive oxygen species (mtROS), hypoxia-inducible factor 1, glycolysis, and EC activation. Inhibition of Drp1 significantly attenuated these phenotypes. Carotid artery ligation and microfluidics experiments further validate that the significant induction of mitochondrial fragmentation was associated with EC activation in a Drp1-dependent manner. Contrarily, UF in vitro or voluntary exercise in vivo significantly decreased mitochondrial fragmentation and enhanced fatty acid uptake and OXPHOS. Our data suggest that flow patterns profoundly change mitochondrial fusion/fission events, and this change contributes to the determination of proinflammatory and metabolic states of ECs.

## Graphical abstract

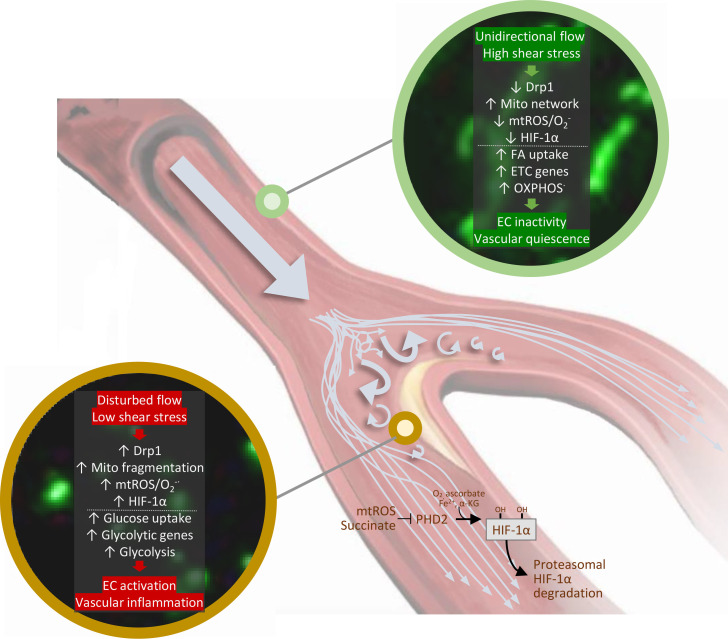

## Introduction

The endothelium is the inner-most layer of the blood vessels and plays a variety of roles in maintaining vascular homeostasis (1). The luminal surface of the endothelium is continually exposed to remarkably dynamic blood flow patterns that often significantly influence immunometabolic phenotypes of the endothelial cells (ECs) (2). For example, it has been shown that unidirectional flow (UF) induces quiescent EC phenotypes including a reduction of glycolysis and an elevation of oxidative phosphorylation–dependent (OXPHOS-dependent) ATP generation (3–6). In contrast, disturbed flow (DF) elicits proinflammatory endothelial activation and increases EC glycolysis with a reduction of OXPHOS (7–9). This transition is a critical step for EC activation such that inhibition of the metabolic reprogramming to glycolysis or enhancement of OXPHOS is considered as an effective strategy to prevent the progression of vascular inflammation (7, 10).

Until recently, the role of mitochondria in ECs was somewhat neglected due to the prevailing view that they were glycolytic cells. However, emerging evidence has shown that endothelial mitochondria are highly active organelles, contributing to their bioenergetic, biosynthetic, and signaling functions (11–14). Indeed, mitochondrial dysfunction has been shown to lead EC apoptosis and EC activation (15). To this end, it is imperative to determine how endothelial mitochondria behave differently under different physiologically occurring flow patterns.

Mitochondrial morphology is dynamically controlled by a balance between fusion and fission processes, and this balance is necessary for maintaining mitochondrial homeostasis (16). Mitochondrial fission is required for mitochondrial quality control by being involved in mitophagy, mitochondrial intracellular distribution, and intact mitochondrial DNA (mtDNA) quality control (17, 18). However, excessive mitochondrial fission has been linked to mitochondrial dysfunction, such as reduced OXPHOS and elevated mitochondrial reactive oxygen species (mtROS) production, which have been associated with various cardiovascular diseases (CVDs) (19–21). Furthermore, CVD risk factors such as hyperglycemia, smoking, oxidized low-density lipoprotein (ox-LDL), and inflammatory mediators, including TNF-α and LPS, are known to induce excessive mitochondrial fission processes in ECs (19, 22, 23).

Drp1 is a cytosolic large GTPase acting as a central player of mitochondrial fission. When activated, Drp1 is translocalized to the outer mitochondrial membrane (OMM), forming an oligomerized ring to constrict a mitochondrial tubule into daughter mitochondria in a guanosine-5’-triphosphate–dependent (GTP-dependent) manner (24). Drp1 has multiple phosphorylation sites that determine its GTPase activity (25). Dephosphorylation at the serine 637 residue is known to increase the Drp1 GTPase activity, resulting in an increase of mitochondrial fission process (26).

Emerging evidence strongly suggests that a regular practice of physical activity, as a nonpharmacological intervention, reduces the progression and development of CVD by improving EC integrity (27), but the precise mechanisms have yet to be completely understood. Studies show that a single bout of exercise significantly elevates magnitude of wall shear stress and eliminates flow oscillation in human arteries, which may link exercise and its beneficial CV effects (28, 29). The purpose of this study was to investigate the effect of different flow patterns on mitochondrial morphology and its implication on EC phenotypes. In this study, we utilized small- and large-animal models, as well as the complementary microfluidics systems, which afford the leverage to provide comprehensive insight into the link between mitochondrial and immunometabolic phenotypes in different flow–exposed ECs.

## Results

### Excessive mitochondrial fragmentation and activation of Drp1 instigate intracellular ROS production and atheroprone endothelial phenotype in the DF-exposed vessel regions.

**Figure 1:**
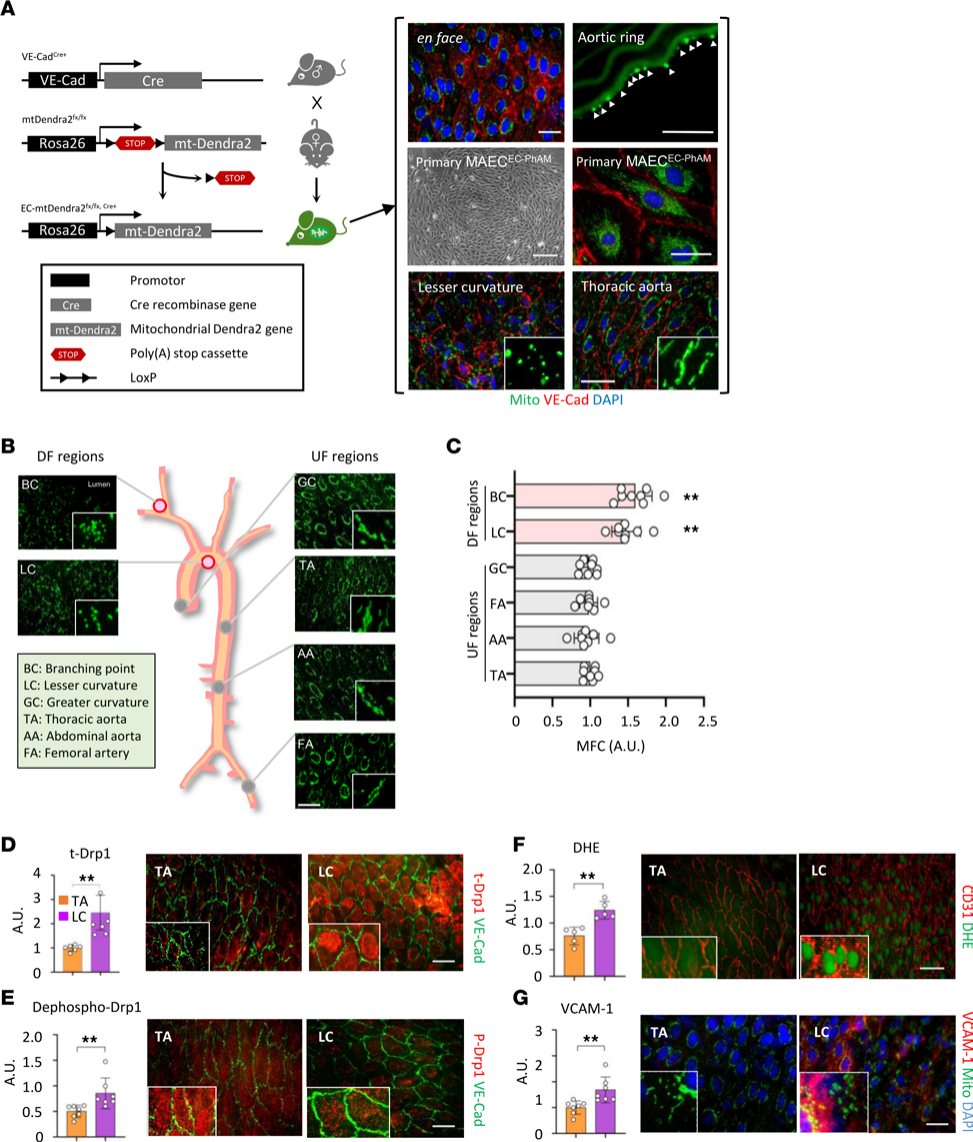
Excessive mitochondrial fragmentation in DF-exposed vessel regions instigates atheroprone endothelial phenotypes in vivo. (**A**) A strategy to generate EC-PhAM mouse (left). In the bracket (right), en face staining, frozen cryosection of aortic ring, phase contrast micrograph of mouse aortic endothelial cells from EC-PhAM mice (MAEC^EC-PhAM^), epifluorescence image of MAEC^EC-PhAM^, en face lesser curvature, and en face thoracic aorta are shown. Mitochondria (green), VE-Cadherin (red), and DAPI (blue) are shown. Scale bar: 200 μm (63× oil lens, phase contrast; middle left) and 20 μm (all others). (**B**) Representative micrographs of mitochondrial morphology at various vessel regions of the arteries. Scale bar: 20 μm. (**C**) Mitochondrial fission count (MFC, mitochondria number/total mitochondria area) (*n* = 8). **P < 0.01. (**D**–**G**) Representative images and plots of each total Drp1 (**D**), dephospho-Drp1 Ser637 (**E**), DHE (**F**), and VCAM-1 (**G**) in the endothelium of lesser curvature (LC, a DF region) and thoracic aorta (TA, a UF region). Scale bar: 20 μm (*n* = 5–7; 63× objective lens). Data are shown as mean ± SD; **P < 0.01 by 1-way ANOVA and Tukey’s post hoc tests (**C**); 2-tailed independent Student’s *t* test (**D**, **F**, and **G**); and Mann-Whitney *U* test (**E**). A.U., arbitrary unit.

To specifically visualize endothelial mitochondria in a mouse tissue, we generated EC-specific photo-activatable mitochondria (EC-PhAM) mice by breeding PhAM-floxed mice with VE-Cadherin–Cre mice (Figure 1A). Briefly, Cre activity excises a poly(A) stop cassette, allowing stochastic expression of mito-Dendra2 specifically in ECs, which is a mitochondrial-specific version of Dendra2 green/red photo switchable monomeric fluorescent protein. As shown in the bracket (Figure 1A), the endothelial-specific mito-Dendra2 signal was confirmed by en face staining, and a frozen aortic ring of the EC-PhAM mice. Primary cultured mouse aortic ECs (MAEC^PhAM^) also showed a typical cobble-stone shape and stable expression of mito-dendra2 signal. Interestingly, the shape of the mitochondria is distinctively different in DF-exposed versus UF-exposed vessel (i.e., lesser curvature [LC] and thoracic aorta [TA], respectively). Using the EC-PhAM mouse, we further examined the morphology of endothelial mitochondria at 6 different vessel segments in vivo, including DF-exposed regions (i.e., LC and bifurcation) and UF-exposed regions (i.e., greater curvature, TA, abdominal aorta, and femoral artery). As shown in Figure 1B, the morphology of endothelial mitochondria in the DF-exposed segments is a shorter fragmented shape, while UF-exposed vessels show elongated mitochondrial network. This is confirmed by mitochondrial fragment counts (MFC) that were significantly higher in the DF-exposed regions compared with UF-exposed vessel regions (Figure 1C). Moreover, LC showed a significantly higher level of total Drp1 expression compared with TA (*P* = 0.002) (Figure 1D) and decreased phosphorylation of Drp1 at Ser637 (*P* = 0.007), an inactivation site of the Drp1 (Figure 1E). In contrast, phosphorylation of the Drp1 Ser616, an activation site, was significantly higher in the LC compared with TA (Supplemental Figure 1, A and B; supplemental material available online with this article; https://doi.org/10.1172/jci.insight.159286DS1). ROS production measured by dihydroethidium (DHE) staining was also significantly elevated at LC than TA (*P* = 0.002) (Figure 1F). DF elevated the expression of NOX4, a primary site for the ROS production in ECs (Supplemental Figure 2), as well as Drp1-dependent mtROS production (see below); cytosolic ROS production measured by DCF staining also showed a significant increase under DF condition, which was mitigated by mdivi-1, a Drp1-specific inhibitor, demonstrating a DF-induced vicious cycle of the increase in the intracellular oxidative stress level (Supplemental Figure 3). Furthermore, in the ECs exposed to DF, 8-OHdG level was significantly increased, demonstrating the oxidative damages in the nuclear and mtDNA (Supplemental Figure 4). As a result, LC showed higher proinflammatory EC activation determined by VCAM-1 expression (*P* < 0.001) (Figure 1G).

### Flow pattern instantaneously, yet reversibly, alters mitochondrial morphology in a Drp1-dependent fashion in primary MAECs.

**Figure 2:**
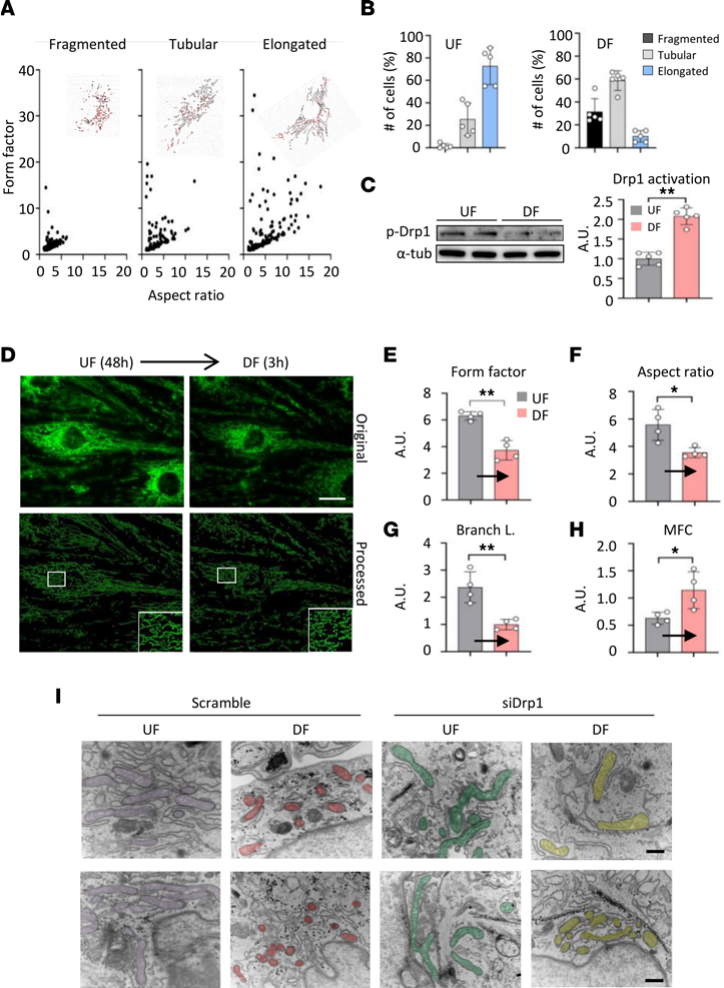
Flow pattern instantaneously but reversibly alters mitochondrial morphology in a Drp1-dependent fashion in primary mouse aortic endothelial cells. (**A**) Mitochondrial morphology classification based on aspect ratio (AR) and form factor (FF). The endothelial mitochondria morphology was classified into 3 subgroups (fragmented, tubular, or elongated) based on AR and FF. (**B**) Quantification plots of mitochondrial morphology under DF versus UF. Primary cultured aortic endothelial cells from EC-PhAM mice (MAEC^PhAM^) were used. (**C**) Representative immunoblot images for the protein expression of phospho-Drp1 at Ser637. α-Tubulin was used as a loading control. Quantification plots of phospho-Drp1 at Ser637 under UF versus DF (*n* = 5). (**D**) Micrographs of endothelial mitochondria under UF and 3 hours after flow transition from UF to DF (*n* = 4). Scale bar: 20 μm. (**E**–**H**) Quantification plots of mitochondrial morphology analyses (AR, FF, and branch length [BL]) under UF and 3 hours after the flow transition from UF to DF. MFC was calculated as number of particles/total area of mitochondria (*n* = 4–6). (**I**) Ultrastructure of mitochondria in HUVECs under UF versus DF. Two sets of representative images are shown. HUVECs were transfected with either scrambled or Drp1 siRNA and subjected to either UF (20 dyne/cm^2^) or DF (5 dyne/cm^2^, 1 Hz) for 48 hours (transition electron microscopy [TEM], 50,000×). Scale bar: 400 nm (*n* = 3). Data are shown as mean ± SD. *P < 0.05, **P < 0.01. by 2-tailed independent Student’s *t* test (**C** and **G**) or Welch’s *t* test (**E**, **F**, and **H**). A.U., arbitrary unit; UF, unidirectional flow; DF, disturbed flow.

To precisely investigate endothelial mitochondrial morphometrics overtime, we isolated primary MAECs from EC-PhAM mice (MAEC^PhAM^), subjected them to either UF (20 dyne/cm^2^) or DF (±5 dyne/cm^2^), and recorded a serial time-lapse image up to 48 hours (Supplemental Figure 5). We observed that EC morphology became gradually elongated in the direction of flow under UF, while ECs under DF appeared rounded and became cobble-stone shaped, which corresponds to the morphology of ECs at TA and LC, respectively (Supplemental Figure 6). Next, based on the mitochondrial morphological classification criteria we established (Figure 2A), we further analyzed mitochondrial shapes. Endothelial mitochondria under UF showed a momentary fragmentation up to ~30 minutes but later showed elongated shapes stably. Mitochondria under DF showed abrupt but continuing changes to more fragmented shapes (Figure 2B) over the DF exposure up to 48 hours. As expected, Drp1 phosphorylation at Ser637 was significantly reduced under DF compared with UF in ECs (*P* < 0.001) (Figure 2C). Of note, a separate experiment using HUVECs showed that no other mitochondrial dynamics–related factors are changed under our flow conditions (Supplemental Figure 2, Supplemental Figure 8, A and B). Next, in order to analyze transient changes in the mitochondrial shapes, we performed a live-cell time-lapse imaging under switching different flow conditions. Initially, MAEC^PhAM^ was exposed to UF for 48 hours following a culture in static flow, and then, the flow pattern was switched to DF. Mitochondria showed elongated morphology after 48 hours of UF, but once the flow was switched to DF, mitochondrial morphology quickly became a fragmented shape (Figure 2D). These changes were confirmed with quantitative morphometrics such as aspect ratio (AR) (*P* = 0.014), form factor (FF) (*P* < 0.001), branch length (BL) (*P* = 0.004), and MFC (*P* = 0.002) (Figure 2, E–H). In addition, fragmented mitochondria under DF were shifted to a more elongated shape after changing flow to UF, and vice versa (Supplemental Figure 5). In order to analyze mitochondria ultrastructure, we transfected HUVECs with a scrambled or Drp1 siRNA and subjected them to either UF (20 dyne/cm^2^) or DF (± 5 dyne/cm^2^, 1Hz) for 48 hours following transmission electron microscopy. As shown in Figure 2I, small, fragmented mitochondria were observed under DF in a Drp1-dependent manner. There are no signs of abnormal cristae structures in any of the endothelial mitochondria.

### Voluntary exercise attenuates mitochondrial fragmentation and VCAM-1 expression in ECs at the DF-exposed vessel regions.

**Figure 3:**
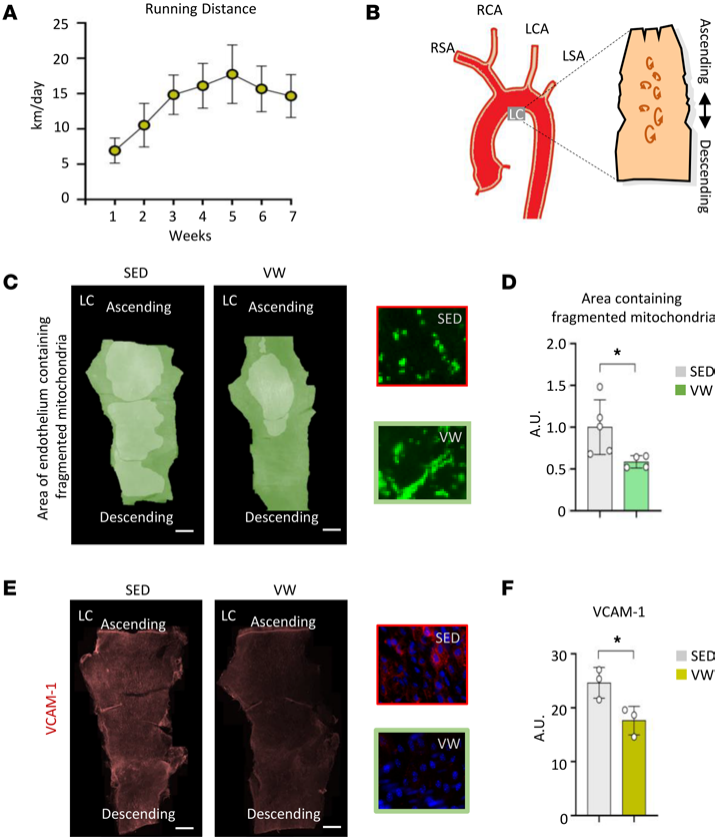
Voluntary wheel exercise attenuates mitochondrial fragmentation and VCAM-1 expression in the endothelium at disturbed flow–exposed vessel regions. (**A**) Average running distance (km/day). (**B**) Illustration of vessel region analyzed. (**C**) Representative stitched micrographs for mitochondrial morphometric analysis. Shaded areas indicate where fragmented mitochondria are observed. (**D**) Quantification plot of the area of endothelium covered by fragmented mitochondria at LC in sedentary versus exercised PhAM mice (*n* = 4–5). (**E**) Representative stitched micrographs of VCAM-1 staining. (**F**) Quantification plot of VCAM-1 expression in sedentary (*n* = 3) versus exercised mice (*n* = 3). Data are shown as mean ± SD. *P < 0.05 by 2-tailed independent Student’s *t* test. LC, lesser curvature; SED, sedentary; VW, voluntary wheel running exercise; RSA, right subclavian artery; RCA, right common carotid artery; LCA, left common carotid artery; LSA, left subclavian artery.

Exercise decreases DF rather promotes UF in the arterial circulation (28–30). To test the effect of voluntary physical activity on mitochondrial fragmentation in ECs, EC-PhAM mice were subjected to either voluntary wheel running (VW) or sedentary conditions (SED) for 7 weeks (Figure 3A), and mitochondrial morphology and VCAM-1 expression were examined. In the LC (Figure 3B), the areas of endothelium-containing fragmented mitochondria were significantly reduced (*P* < 0.044) (Figure 3, C and D), and VCAM-1 expression was significantly reduced in the VW compared with SED (*P* < 0.036) (Figure 3, E and F), suggesting that physiological alteration of the flow patterns mitigates mitochondrial fragmentation at the atheroprone regions.

### DF causes excess mitochondrial fragmentation and vascular inflammation in a carotid artery partial ligation model.

**Figure 4:**
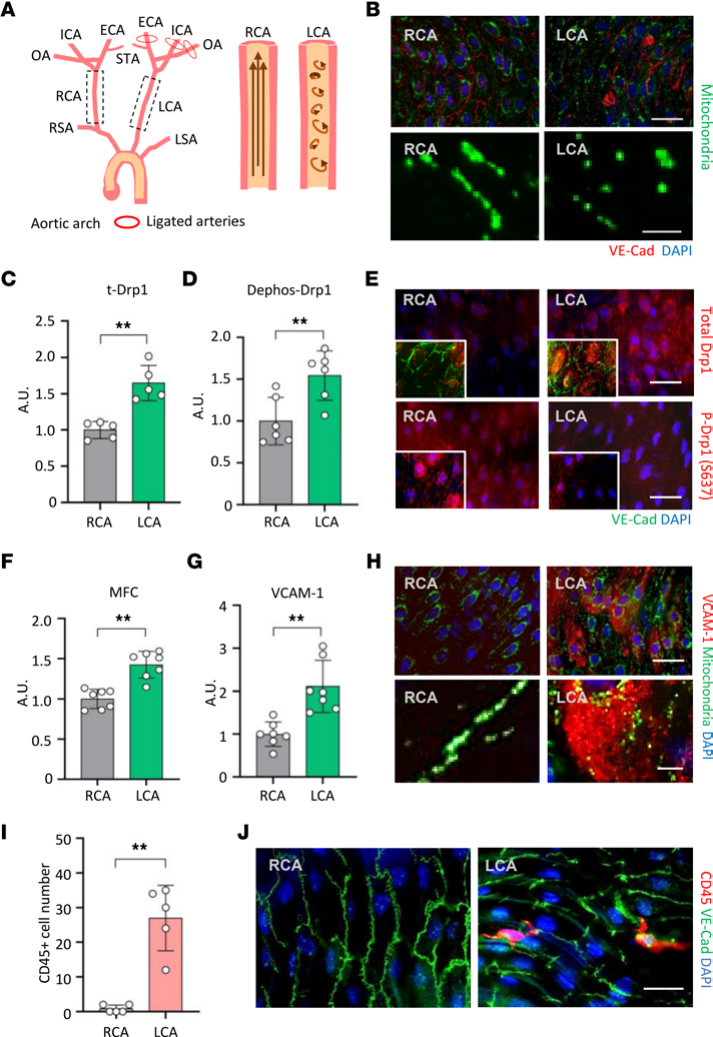
DF causes excess mitochondrial fragmentation and vascular inflammation in a carotid artery partial ligation model. (**A**) Schematic illustration of ligation sites. ECA, external carotid artery; ICA, internal carotid artery; OA, occipital artery; STA, superior thyroid artery; RSA, right subclavian artery; LSA, left subclavian artery; RCA, right common carotid artery; LCA, left common carotid artery. (**B**) Representative fluorescence images of endothelial mitochondria in LCA versus RCA after 48 hours of partial ligation surgery (63×). Scale bar: 30 μm (top), 3 μm (bottom). (**C**) Quantification plot of total Drp1 in RCA versus LCA (*n* = 5). (**D**) Quantification plot of Dephos-Drp1 at Ser637 in RCA versus LCA (*n* = 6). (**E**) Representative micrographs of total Drp1 and phospho-Drp1 at Ser637 in RCA versus LCA. Scale bar: 30 μm. (**F**) Mitochondrial fission count (MFC) (*n* = 7). (**G**) Quantification plot of VCAM-1 expression (*n* = 7). (**H**) Representative images of VCAM-1 expression in LCA versus RCA after 48 hours of partial ligation (63×). Scale bar: 30 μm (top), 5 μm (bottom). (**I**) Bar graph shows the average number of CD45^+^ cells in a given aortic ring section (3 mm). (**J**) Representative fluorescence images of CD45^+^ cells and VE-Cadherin in the en face of LCA versus RCA. Scale bar: 30 μm. Data are shown as mean ± SD. **P < 0.01 by 2-tailed independent Student’s *t* test (**C**, **D**, **F**, and **G**) or Mann-Whitney *U* test (**I**). A.U., arbitrary unit.

To further test whether pathophysiological DF induces mitochondrial fragmentation in vivo, we employed a carotid artery partial ligation model in the EC-PhAM mice (31). As shown in Figure 4A, 3 of the 4 upper branches of the left carotid artery (LCA) were ligated, while the superior thyroid artery was left open. This procedure has been shown to significantly elevate turbulent flow in the carotid artery and causes vascular inflammation. In this model, the intact right carotid artery (RCA) served as an internal control (Figure 4A). En face preparation of the LCA showed a significantly more fragmented mitochondria compared with the RCA (Figure 4B), which is also evidenced by increased MFC analysis (*P* < 0.001) (Figure 4F). Also, the LCA showed higher total Drp1 expression (*P* < 0.001), as well as increased dephosphorylation at Ser637 (*P* = 0.009) (Figure 4, C–E) and phosphorylation at Ser 616 (Supplemental Figure 1, C and D) compared with the intact RCA, indicating Drp1 activation. The LCA simultaneously showed elevation of VCAM-1 expression (*P* < 0.001) (Figure 4, G and H). As shown in the Figure 4, I and J, en face preparation of the LCA showed significantly higher numbers of inflammatory leukocyte adhesion (CD45^+^) compared with the RCA, suggesting vascular inflammation.

### Flow pattern alters immunometabolic phenotypes of ECs in a Drp1-dependent manner.

**Figure 5:**
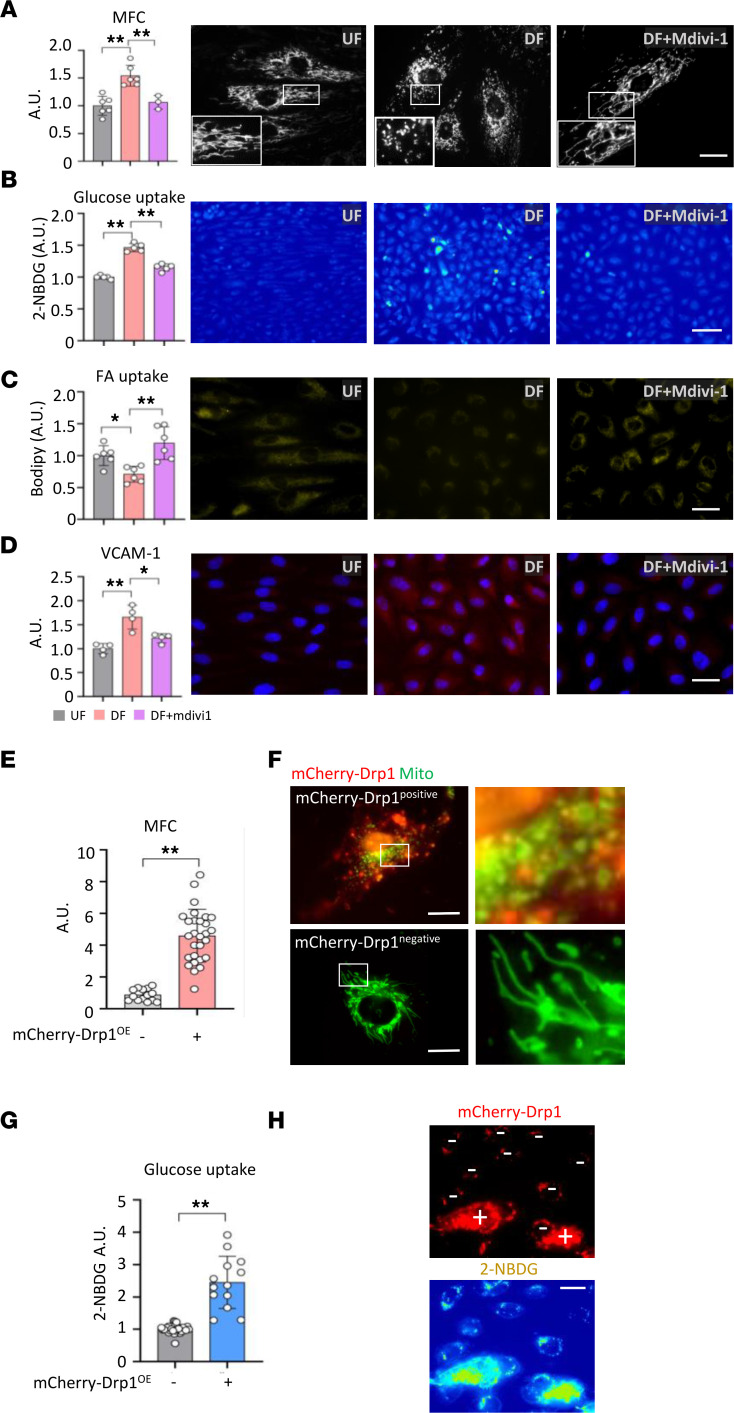
Flow pattern alters immunometabolic phenotypes in endothelial cells in a Drp1-dependent manner. (**A**) Representative fluorescence images of mitochondria morphology in MAEC^PhAM^ under UF versus DF versus DF + Mdivi1 (25 μM). Scale bar: 30 μm (*n* = 3–6). (**B**) Representative fluorescence images of 2-NBDG uptake in HAECs under UF versus DF versus DF + Mdivi1 (25 μM). Scale bar: 100 μm (*n* = 4) (**C**) Representative fluorescence images of fatty acid (BODIPY) uptake in HAECs under UF versus DF versus DF + Mdivi1 (25 μM). Scale bar: 30 μm (*n* = 6). (**D**) Representative fluorescence images of VCAM-1 expression in HAECs under UF versus DF versus DF + Mdivi1 (25 μM). Scale bar: 30 μm (*n* = 4). (**E**) Quantification plot of mitochondrial fission count (MFC) in mCherry-Drp1 overexpression vector–positive or –negative HUVECs. (**F**) Representative micrographs of mCherry-Drp1 (red) and mitochondria stained with MitoTracker Green FM (green). Scale bar: 30 μm. (**G**) Quantification plot of 2-NBDG intensity in mCherry-Drp1 overexpression vector–positive or –negative HUVECs. (**H**) Representative fluorescence images of mCherry-Drp1 (red) and 2-NBDG glucose uptake (yellow). Scale bar: 30 μm. Data are shown as mean ± SD. *P < 0.05, **P < 0.01 by Welch’s *t* test (**E**), Mann-Whitney *U* test (**G**), or 1-way ANOVA followed by Tukey’s post hoc test (**A**–**D**). A.U., arbitrary unit; UF, unidirectional flow; DF, disturbed flow.

Because of the difficulty of obtaining sufficient protein sample from mouse models, we obtained canine aorta to collect protein lysates from each LC and TA in the aorta for downstream experiments (Supplemental Figure 7A). Similar to mouse models above, the canine aorta showed a significant increase in total Drp1 protein at LC (*P* < 0.001). Furthermore, DF-exposed regions of the aorta showed increased rate-limiting glycolytic enzymes (e.g., hexokinase 2 [HK2; *P* = 0.028] and pyruvate dehydrogenase 1 [PDK1; *P* < 0.001], compared with TA) (Supplemental Figure 7, B–E). Moreover, to test the effect of different flow patterns on endothelial metabolic profile, we assessed glucose and fatty acid uptake. Treatment of MAEC^PhAM^ with mdivi-1 showed a dramatic decrease in the mitochondrial fragmentation under DF condition, yet it was unchanged in the cell size and cell morphology (Figure 5A). DF significantly elevated glucose uptake (*P* < 0.001) and decreased fatty acid uptake (*P* = 0.042) (Figure 5, B and C). DF also significantly elevated VCAM-1 expression levels (*P* < 0.001) (Figure 5D). Inhibition of Drp1 activity by mdivi-1 significantly attenuated glucose uptake and VCAM-1 expression and restored DF-induced reduction of the fatty acid uptake (*P* = 0.036) in ECs. To test whether Drp1 is sufficient to carry out the mitochondrial elongation and the DF-mediated glucose metabolism, we transiently transfected HUVECs with a mCherry-Drp1 overexpression construct and examined the uptake of metabolic substrate. As shown in Figure 5, E–H, mCherry-Drp1 overexpression significantly increase the fragmentation of mitochondria and elevated glucose uptake.

**Figure 6:**
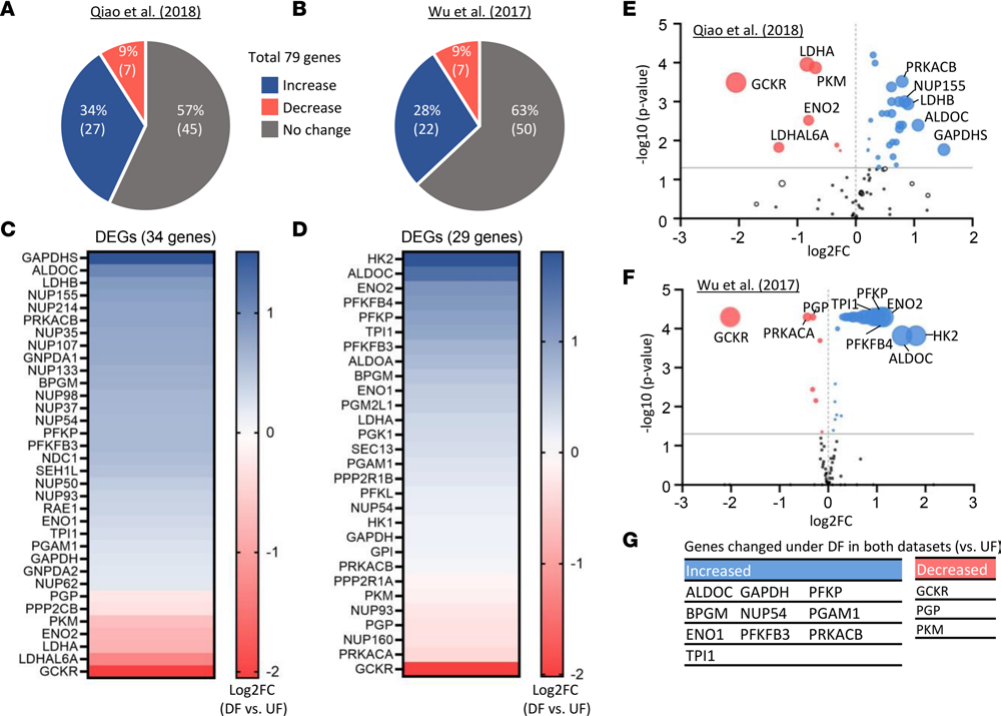
Analysis of RNA-Seq database for glycolysis-related genes. (**A** and **B**) Numbers and percentages of the total of 79 glycolytic genes that are increased, decreased, or unchanged (DF versus UF). (**C**–**F**) Heatmaps and volcano plots presenting genes expression profile of more than log_2_ fold change in the Qiao et al. (2018) (32) (**C** and **E**) and Wu et al. (7) (**D** and **F**). The dot size of the volcano plots represents the product of log_2_fold change and −log_10_ *P* values. DEGs, differentially expressed genes. (**G**) The most-changed metabolic genes (DF versus UF).

To further examine the effect of different flow patterns on global metabolic profile in ECs, we conducted in silico transcriptome analysis of RNA-Seq data sets from 2 published papers (7, 32), which revealed an increment of mRNA expressions related to glycolysis under DF compared with UF in ECs (Figure 6, A–G).

### DF-induced mitochondrial fragmentation increases HIF-1α nuclear localization.

**Figure 7:**
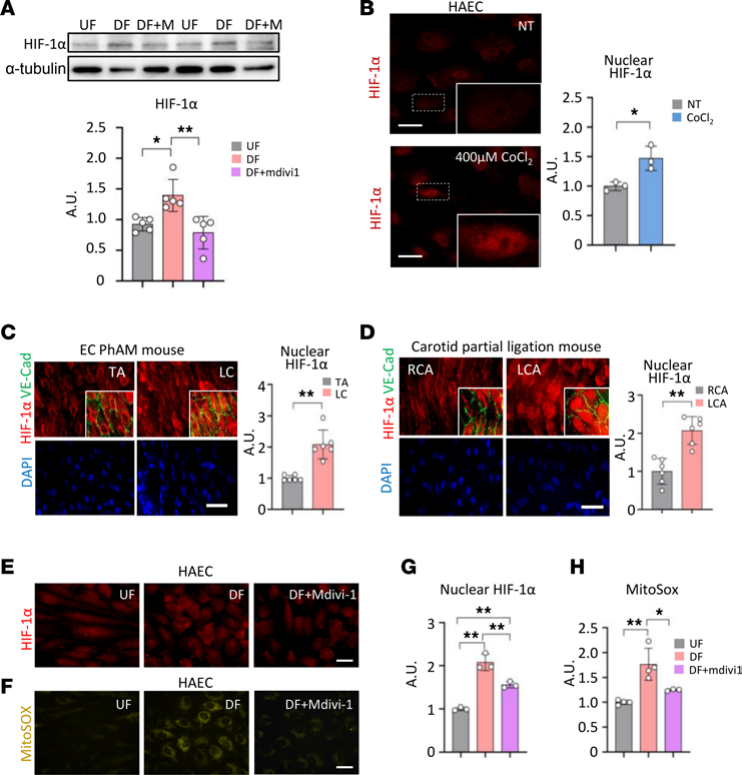
DF induces HIF-1α nuclear localization and increased mtROS production. (**A**) Immunoblot analysis of HIF-1α protein expression under different flow patterns in HAECs. α-Tubulin was used as a loading control (*n* = 5). (**B**) Optimization of HIF-1α nuclear localization. Scale bar: 30 μm (*n* = 3). (**C**) En face staining of nuclear HIF-1α in EC-PhAM mouse aorta (*n* = 6). Scale bar: 30 μm. TA, thoracic aorta; LC, lesser curvature of aortic arch. (**D**) En face staining of nuclear HIF-1α in carotid artery ligation mouse model (*n* = 6). Scale bar: 30 μm. RCA, right common carotid artery; LCA, left common carotid artery. (**E** and **G**) Representative fluorescence images and plot of nuclear HIF-1α under UF, DF, or DF + mdivi1 conditions in HAECs (*n* = 3). Scale bar: 30 μm. (**F** and **H**) Representative fluorescence images and plot of mitochondrial superoxide production measured by mitoSOX probe under UF, DF, or DF + mdivi1 conditions in HAECs (*n* = 3–4). Scale bar: 30 μm. Data are shown as mean ± SD. *P < 0.05. **P < 0.01 by 2-tailed independent Student’s *t* test (**B** and **D**), Mann-Whitney *U* test (**C**), 1-way ANOVA followed by Tukey’s post hoc test (**G** and **H**), or Kruskal-Wallis test followed by Dunn’s test (**A**). A.U., arbitrary unit; UF, unidirectional flow; DF, disturbed flow; DF+M, disturbed flow + mdivi1.

HIF-1α has been shown as an important molecular mediator for glucose metabolism (7). HIF-1α expression level was increased under DF, but this change was significantly attenuated when treated with mdivi-1 (Figure 7A). As for optimizing measurement of nuclear HIF-1α, we tested cobalt chloride–induced (CoCl_2_-induced) HIF-1α activation in human aortic ECs (HAECs) (Figure 7B). DF showed a significantly higher level of nuclear localized HIF-1α (Figure 7C). Furthermore, a similar increase of nuclear HIF-1α was observed in DF-inducing ligated arteries compared with the control artery in the carotid artery partial ligation mouse model described above (Figure 7D). We further examined the role of Drp1 on DF-induced HIF-1α activation in HAECs. DF increased nuclear HIF-1α levels (Figure 7, E and G) and mtROS production (*P* = 0.002) (Figure 7, F and H) in a Drp1-dependent fashion.

## Discussion

In the present study, we demonstrate that mitochondrial morphology is altered by the pattern of flow exposed to the ECs, and this pattern is associated with inflammatory and metabolic phenotypes. This study is the first to show, to the best of our knowledge, that the DF-induced mitochondrial fragmentation is associated with EC activation and EC dysfunction. Our findings also show that voluntary physical exercise significantly attenuated EC activation specifically at the DF-exposed vessel region. In order to address the flow-dependent endothelial mitochondrial morphological alteration, we employed an EC-PhAM mouse model that made the study capable of observing this phenomenon at the tissue level in vivo. These in vivo findings were confirmed by a complementary in vitro microfluidics model and voluntary training experiments.

Our findings with small (mouse) and large (canine) animals and in vitro models provide comprehensive, time-dependent evidence of the flow pattern–dependent endothelial mitochondrial remodeling. There are 2 seemingly contradicting results in the literature, in which Breton-Romero et al. (33) show that UF initially induces transient mitochondrial fragmentation at early phase (30 minutes) (33), whereas Wu et al. (2018) reported increased elongated endothelial mitochondria under prolonged UF exposure (6–12 hours) (18). These temporal responses of endothelial mitochondrial shape to UF are consistent with what we observed using time-lapse live-cell imaging. Furthermore, the biphasic temporal responses of EC to UF are somewhat similar to what has been reported previously by Hahn and Schwartz (34). Their study described that UF transiently induces various inflammatory signaling pathways, such as an increase of ROS production and expression of proinflammatory mediators such as c-JUN N-terminal kinase, NF-κB, and p21-activated kinase; however, these responses were downregulated within 1 hour. In contrast, DF activates similar inflammatory pathways in a sustained manner, leading to an activated EC phenotype (34).

In this study, we observed a casual relationship between mitochondrial fragment and the EC immunometabolic phenotype, as shown in Figure 5. For example, we showed that inhibition of mitochondrial fragmentation by mdivi-1 significantly attenuated DF-induced glucose uptake, VCAM-1 expression, and ROS production and increased fatty acid uptake. These findings are in alignment with recent studies by other groups showing that metabolic transition from OXPHOS to glycolysis is a crucial step for EC to initiate proinflammatory activation (7, 8). In addition, Xiao et al. (10) demonstrated that stimulation of glycolysis promotes EC inflammation, whereas enhancement of OXPHOS suppresses inflammation in ECs upon encountering inflammatory stimulation. This notion is also well aligned with our high-throughput and quantitative transcriptome profiling analyses, as shown in Figure 6. Future studies are warranted to further investigate the fate of metabolites and the consequences of the metabolic shift on the entire cell bioenergetics and biosynthetic function.

Considering the relationship between mitochondrial dynamics and cell metabolism, several studies previously reported the connection under various physiological. Parra et al. (35) reported that inhibition of mitochondrial fission prevented metabolic shift toward glycolysis during hypoxia, which reduced proliferation and apoptosis in vascular smooth muscle cells (VSMC). Buck et al. (36) reported that mitochondrial morphology determines T cell fate through metabolic reprogramming to be activated effector T cells (T_E_) or quiescent memory T cells (T_M_). This study showed that T_E_ contains fragmented mitochondria showing reduced electron transport chain (ETC) efficiency and enhanced glycolysis, while T_M_ carries elongated mitochondria shape, accompanied by efficient ETC complex associations and favoring OXPHOS. Together, these studies demonstrate potential mechanisms by which mitochondrial structural changes cause metabolic cascades in the cells.

In the present study, we observed an elevation of Drp1 expression/activity concomitant with mitochondrial fragmentation under DF, and inhibition of Drp1 activity by mdivi-1 attenuated DF-induced atheroprone EC phenotype. With regard to the clinical implication of these findings, Wang et al. (37) show that hyperglycemia-induced mitochondrial fragmentation in ECs is associated with atherosclerotic lesion formation in diabetic ApoE^–/–^ mice. In this study, the inhibition of mitochondrial fission by mdivi-1 treatment attenuated hyperglycemia-accelerated atherosclerosis by diminishing cellular oxidative stress level. The implication of the relationship between mitochondrial fragmentation and vascular inflammation can be further investigated using a genetically modified fragmented mitochondria model (e.g., EC-Drp1 transgenic mouse) combined with an atherosclerotic mouse model.

Precise molecular mechanisms underlying the mitochondrial-phenotypic relationships were out of the scope of the current study. However, we postulated that the regulation of cellular phenotype by mitochondrial fusion/fission dynamics could be explained in 2 ways. Firstly, previous studies show that mitochondrial morphology is highly relevant to its respiratory capacity. Extended mitochondrial network via elongation increases OXPHOS due to efficient Ca^2+^ supply from endoplasmic reticulum via mitochondria-associated endoplasmic reticulum membranes and mitochondrial calcium uniporter, in that mitochondrial Ca^2+^ is an essential second messenger for the tricarboxylic acid (TCA) cycle. For example, Ca^2+^ activates 3 dehydrogenases in the TCA cycle, including pyruvate dehydrogenase, NAD^+^-dependent isocitrate dehydrogenase, and 2-oxoglutarate dehydrogenase, such that low Ca^2+^ level in mitochondria decreases the rate of TCA cycle (38). In addition, mitochondrial fusion has been shown to be required for fatty acid distribution and oxidation that ensures maximum oxidative metabolism (39). An elongated mitochondrial network is associated with an efficient respiratory chain supercomplex associated with enhanced OXPHOS (36). Based on the reasons described above, disconnected mitochondrial network has been associated with impaired OXPHOS (40), showing a reduction of mitochondrial respiration by 20%–80% with higher cellular apoptosis rate (41). Secondly, reduced OXPHOS in fragmented mitochondria may alter retrograde signaling patterns, a pathway of communication from mitochondria to the nucleus, by altering the generation of TCA cycle intermediates or mtROS production (42). More studies are warranted to determine the exact mechanisms underlying how the morphological change of mitochondria modulates the phenotypic changes in ECs, what the molecules are, and its downstream signaling pathways that mediate mitochondrial retrograde signaling.

There may be some limitations in this study. First, we were unable to conduct the Seahorse bioanalyzer–based endothelial mitochondrial OCR and extracellular acidification rate (ECAR) analyses due to a technical limitation of conducting the assays under different flow conditions. However, we previously reported that laminar flow increases mitochondrial oxygen consumption in HUVECs using a Clark-type polarographic dissolved oxygen probe, while a static flow was used as a control (43). We also observed that laminar flow increases gene expression related to TCA cycle (unpublished data). Furthermore, in the present study, we showed gene expression profile under different flow conditions, which is consistent with other findings of the DF-induced enhancement of glycolytic pathway. Second, the loss-of-function study using EC-Drp1–KO mice or gain-of-function study using EC-Drp1–overexpression mice was not conducted; these studies would provide more insightful data to understand a causal relationship between Drp1-mediated mitochondrial morphology and EC phenotypes in vivo. Future studies are warranted to test whether Drp1-dependent metabolic switch occurs in vivo. Third, our data show changes of Drp1 downstream events under different flow conditions, including mtROS, HIF-1α, glucose and fatty acid uptake, and EC activation, although the precise underlying mechanisms and relationships between the events remain to be explored.

Taken together, our data provide potentially novel insights into the molecular mechanisms of mitochondria-dependent alternations in endothelial phenotypes under different flow conditions. The present study demonstrates that atheroprone phenotype under oscillatory DF was facilitated by Drp1-dependent mitochondrial fragmentation. In addition, our data suggest that regular engagement in physical activity may decrease atheroprone-phenotypes in ECs, especially in the DF-exposed vessel regions, through a potential mechanism mediated by mitochondria as mechanosensing intracellular organelles. Future studies are warranted to better understand underlying mechanisms for the intersection between mechanobiology, mitochondrial dynamics, and EC activation.

## Methods

### Animals.

PhAM-floxed mice (B6;129S-Gt[ROSA]26Sor^tm1[CAG-COX8A/Dendra2]Dcc^/J) and VE-Cadherin–Cre mice (B6;129-Tg[Cdh5-cre]1Spe/J) were purchased from The Jackson Laboratory. EC-PhAM mice was generated following the method presented in a previous study (44). All mice were fed a chow diet and water ad libitum under a 12-hour light/dark cycle. Mice were anesthetized with isoflurane and euthanized by cervical dislocation. For canine aorta samples, animals were euthanized via Euthasol (390 mg/mL pentobarbital sodium and 50 mg/mL phenytoin sodium) at 1 mL/10 lb, i.v. injection. Freshly isolated canine aortas were obtained from one-year-old male dogs (*n* = 3). Detailed methods for canine aorta collection and EC isolation procedure are provided in Supplemental Methods.

### Blood vessel isolation and en face immunostaining.

Mice were anesthetized with isoflurane and then perfused with 10 mL of cold PBS followed by perfusion with a fixative (10 mL of cold 2% paraformaldehyde [PFA]). Different regions of the aortas and arteries, including the aortic arch, carotid artery, TA, abdominal aorta, femoral artery, and mesenteric artery, were isolated. The isolated blood vessels were postfixed with 0.4% PFA overnight at 4°C. The PFA-fixed vessels were subjected to en face immunostaining following the method previously described (43). Images were acquired under a fluorescence microscope (AxioImager, Zeiss) with the 20× and 63× oil objective lens. Detailed methods are provided in Supplemental Methods.

### Mitochondrial morphology quantification.

A quantitative analysis of mitochondrial morphology was performed based on the methods described previously (45). Briefly, obtained images were processed using ImageJ (NIH) to subtract backgrounds and subjected to kernel convolution to emphasize the edges of each mitochondrial particle. Using the binary images, mitochondrial segments were identified and counted with analyze particles function of ImageJ, and the number was normalized to the total mitochondrial area to obtain the mitochondrial fragmentation count (MFC) for each imaged cell (MFC = mitochondria number/total mitochondrial area). Detailed methods are provided in Supplemental Methods.

### Immunoblotting.

Immunoblotting was performed as described previously (43). Briefly, protein samples were subjected to SDS-PAGE and transferred to a polyvinylidene difluoride membrane probed with the following antibodies: Drp1 (BD Biosciences, 610296), CD144 (Invitrogen, 14-1441-81), p–Drp1 Ser637 (Cell Signaling Technology, 6319), β-actin (Sigma-Aldrich, A1978), VCAM-1 (Santa Cruz Biotechnology Inc., sc-13160), CD31 (MilliporeSigma, MAB1398Z), PDK1 (Santa Cruz Biotechnology Inc., sc-515944), HK2 (Santa Cruz Biotechnology Inc., sc-374091), T-eNOS (BD Biosciences, 610296), OPA1 (BD Biosciences, 612606), Mfn2 (Santa Cruz Biotechnology Inc., sc-100560), Fis1 (Sigma-Aldrich, HPA017430), and α-tubulin (Sigma-Aldrich, T9026). Detailed methods are provided in the Supplemental Methods.

### Carotid artery partial ligation.

Partial ligation of the LCA was carried out to generate artificial DF as previously described (31). Briefly, mice were anesthetized by an i.p. injection of a xylazine (10 mg/kg) and ketamine (80 mg/kg) mixture with saline. The incision area was disinfected with betadine, and a ventral midline incision (4–5 mm) was made in the neck. The LCA was exposed by blunt dissection, and then 3 of the 4 LCA branches (left external carotid, internal carotid, and occipital artery) were ligated with a 6–0 silk suture**,** while the superior thyroid artery was left intact (Figure 4A). The incision was then closed with interrupted sutures. After the surgery, mice were monitored until recovery in a chamber on a heating pad. Forty-eight hours later, the mice were sacrificed for further analysis.

### Cell culture and laminar flow applications.

HAECs were purchased from Lonza cultured in M199 medium supplemented with 20% FBS and EC growth supplement and maintained at 37°C in a 5% CO_2_ atmosphere. All experiments with HAECs were conducted between 5 and 9 passages. Cells were exposed to either UF (20 dyne/cm^2^) or disturbed oscillatory laminar flow (DF, ± 5 dyne/cm^2^, 1 Hz) for 48 hours using an Ibidi in vitro flow system (Ibidi) once cells reached 100% confluency. The perfusion sets and fluidic units were kept and operated in a 37°C and 5% CO_2_ incubator. Detailed methods are provided in Supplemental Methods.

### Primary MAEC isolation and live cell imaging.

Primary MAECs were isolated from the aorta of EC-PhAM mice following the protocol described previously (46). Live cell imaging was performed for measuring mitochondrial morphology. Primary cultured MAECs from EC-PhAM mice (MAEC^PhAM^) were seeded into Ibidi μ-slides and exposed to either UF or DF for 48 hours using Ibidi pump system. Temperature and CO_2_ were maintained at 37°C and 5% CO_2_ by a stage-top incubation system (Ibidi). Images were acquired using an epifluorescence inverted microscope (ZEISS AxioVert.A1) with a 20× or 63× objective oil lens. Detailed methods are provided in Supplemental Methods.

### Glucose and fatty acid uptake assays.

HAECs were seeded in μ-slides (Ibidi) and subjected to either UF or DF for 48 hours. Immediately after the flow applications, cells were subjected to glucose and fatty acid uptake assays using 2-NBDG glucose uptake assay kit (K682-50, BioVision) and BODIPY fatty acid probe (5 μM, C1-BODIPY 500/510 C12, D3823, Molecular Probes), respectively. Detailed methods are provided in Supplemental Methods.

### mtROS measurement.

Mitochondrial superoxide production was visualized in cultured HAECs using the fluorescent probe MitoSox Red (M36008, Molecular Probes), which has an excitation/emission of 510/580 nm. After removing medium and rinsing 3 times with prewarmed PBS, HAECs were incubated with 5 μM of MitoSox probe for 10 minutes in prewarmed media at 37°C. HAECs were rinsed with prewarmed PBS 3 times and then subjected to live-cell imaging under a fluorescence microscope. ImageJ (NIH) was used for the quantification of fluorescent intensity.

### Expression levels of glycolytic genes in publicly available RNA-Seq data sets.

Expression levels of glycolytic genes were determined in RNA-Seq datasets from refs. 7 and 32 (GSE83476). Lists of the genes involved in glycolysis were obtained from online databases of biological pathways, including the Kyoto Encyclopedia of Genes and Genomes (KEGG; Glycolysis - Homo sapiens; N00731) and Reactome (Glycolysis - Homo sapiens; R-HAS-70171.6), and mRNA expression levels of a total of 79 genes were evaluated in the data sets. For the data set of Qiao et al. (32), Genevestigator software (NEBION AG) was used to check mRNA expression level of genes in the list. In the study, a cone-and-plate apparatus was utilized to apply either 15 dyne/cm^2^ UF or 5 dyne/cm^2^ DF (1 Hz) on human coronary artery ECs (HCAECs) for 24 hours. For the data set of Wu et al., the normalized data set provided in Supplemental Methods was utilized for further analyses (18). In the study, either UF or DF was applied to HAECs for 24 hours.

### VW.

After 3 days of acclimation, EC-PhAM mice were randomly assigned to either SED or VW exercise (VW) group. VW group animals were individually housed in a rat-sized cage with a metal wheel with a diameter of 11.5 cm fitted with a digital magnetic counter for running-distance measurement. SED group animals were singly housed in the same sized cage without the running wheel. All animals were given water and food ad libitum under a 12-hour light/dark cycle. Exercise began at 8 weeks old and continued for 7 weeks as previously described (47).

### Statistics.

Statistical analysis was performed using SPSS software version 18.0 (IBM). The results are presented as mean ± SD. The Shapiro-Wilk normality test and Levene’s test were conducted to measure normal distribution of dependent variables and equal variance between groups, respectively (Supplemental Table 1). For normally distributed data with equal variance, depending on how many conditions were compared, either 2-tailed independent *t* test analysis or 1-way ANOVA with Tukey’s post hoc analysis was conducted. For data that did not pass the normality test, nonparametric tests including the Mann-Whitney *U* test or the Kruskal-Wallis test, followed by the Dunn’s test, were conducted to examine statistical significance between 2 experimental conditions or for experiments with ≥ 3 groups, respectively. For normally distributed data with unequal variance, the Welch’s2-tailed *t* test or the Welch’s 1-way ANOVA was used to examine statistical significance between 2 or ≥ 3 experimental conditions, respectively. *P* < 0.05 was considered statistically significant for all analyses.

### Study approval.

All animal experiments were approved by the IACUC (no. 5090 for mouse; no. 4765 for canine) of Temple University and conformed to *Guide for the Care and Use of Laboratory Animals* (National Academies Press, 2011).

## Author contributions

SGH and JYP conceived the study. SGH, J Shin, SYC, BMM, and JCP obtained the data. RT, FAR, MDB, XY, and JYP participated in data interpretation and discussed the results. SGH primarily drafted the manuscript and constructed figures. J Sayoc and JSS provided critical assistance in manuscript preparation. FAR and JYP edited the manuscript. All authors edited the manuscript, and all authors approved the final submitted version.

## Supplementary Material

Supplemental data

## Version 1 09/22/2022

Electronic publication

## Version 2 09/27/2022

Affiliation correction on fourth location

## Version 3 10/03/2022

Added a missing affiliation for Fabio A. Recchia
