# Supplementary material for: Flow pattern–dependent mitochondrial dynamics regulates the metabolic profile and inflammatory state of endothelial cells
Source: JCI Insight. 2022 Sep 22;7(18):e159286. doi: 10.1172/jci.insight.159286 (PMC9514384; doi:10.1172/jci.insight.159286)
Supplement: Supplemental data [file jciinsight-7-159286-s248.pdf]

Supplementary Data

Short title: Flow pattern-dependent mitochondrial dynamics

Flow pattern-dependent mitochondrial dynamics regulates metabolic profile and inflammatory state of endothelial cells

Soon-Gook Hong[1,2], Junchul Shin[1], Soo Young Choi[1], Jeffery C. Powers[1], Benjamin M. Meister[1,2], Jacqueline Sayoc[1], Jun Seok Son[3], Ryan Tierney[2], Fabio A. Recchia[1,4], Michael D. Brown[5], Xiaofeng Yang[1], and Joon-Young Park[1,2,6]

[1]*Cardiovascular Research Center, Lewis Katz School of Medicine, Temple University, Philadelphia, Pennsylvania, U.S.A.*
[2]*Department of Kinesiology, College of Public Health, Temple University, Philadelphia, Pennsylvania, U.S.A.*
[3]*Laboratory of Perinatal Kinesioepigenetics, Department of Obstetrics, Gynecology and Reproductive Sciences, University of Maryland School of Medicine, Baltimore, Maryland, U.S.A.*
[4]*Institute of Life Sciences, Scuola Speriore Sant'Anna, Pisa, Italy*
[5]*Department of Kinesiology, School of Public Health, University of Maryland, College Park, Maryland, U.S.A.*
[6]*Robbins College of Human Sciences, Baylor University, Waco, Texas, U.S.A.*

Correspondence to Joon-Young Park, Ph.D., 1312 S 5th Street, One Bear Place #97313, Baylor University, Waco, TX 76798-7313, USA. Phone: +1- 254-710-3505; E-mail: joon_park@baylor.edu

Supplemental Table S1

Table S1. Results of normality and equal variance tests

			Normality		Equal variance			
Fig	Sub-Fig	n/group	Shapiro-Wilk test (p-value)		Levene's test (p-value)		Statistical test	Post-hoc test
1	C	8	TA: 0.237, AA: 0.402, FA: 0.543, GC, 0.399, LC: 0.443, BC: 0.796	Yes	0.275	Yes	One-way ANOVA	Tukey’s test
	D	6	TA: 0.559, LC: 0.054	Yes	0.107	Yes	Independent t-test	
	E	7	TA: 0.262, LC: 0.029	No	0.136	Yes	Mann-Whitney U Test	
	F	5	TA: 0.348, LC: 0.341	Yes	0.999	Yes	Independent t-test	
	G	7	TA: 0.562, LC: 0.063	Yes	0.15	Yes	Independent t-test	
2	C	5	RCA: 0.151, LCA: 0.456	Yes	0.121	Yes	Independent t-test	
	D	6	RCA: 0.095, LCA: 0.791	Yes	0.271	Yes	Independent t-test	
	F	7	RCA: 0.109, LCA: 0.323	Yes	0.297	Yes	Independent t-test	
	G	7	RCA: 0.911, LCA: 0.152	Yes	0.061	Yes	Independent t-test	
	I	5	RCA: 0.006, LCA:0.310	No	0.022	No	Mann-Whitney U Test	
3	D	4-5	SED: 0.567, EX: 0.073	Yes	0.101	Yes	Independent t-test	
	F	3	SED: 0.579, SED: 0.336	Yes	0.933	Yes	Independent t-test	
4	C	5	UF: 0.188, DF: 0.924	Yes	0.089	Yes	Independent t-test	
	E	4	UF: 0.777, DF: 0.645	Yes	0.036	No	Welch's t-test	
	F	4	UF: 0.901, DF: 0.105	Yes	0.043	No	Welch's t-test	
	G	4	UF: 0.858, DF: 0.360	Yes	0.136	Yes	Independent t-test	
	H	4	UF: 0.743, DF: 0.819	Yes	0.029	No	Welch's t-test	
5	A	3-6	UF: 0.904, DF: 0.215, DF+M: 0.922	Yes	0.52	Yes	One-way ANOVA	Tukey’s test

	B	5	UF: 0.412, DF: 0.760, DF+M: 0.622	Yes	0.181	Yes	One-way ANOVA	Tukey's test
	C	6	UF: 0.813, DF: 0.342, DF+M: 0.328	Yes	0.056	Yes	One-way ANOVA	Tukey's test
	D	4	UF: 0.420, DF: 0.859, DF+M: 0.136	Yes	0.214	Yes	One-way ANOVA	Tukey's test
	E	14-28	mChe-Drp1-: 0.133, mChe-Drp1+: 0.811	Yes	0.001	No	Welch's t-test	
	G	15-34	mChe-Drp1-: 0.004, mChe-Drp1+: 0.904	No	0.001	No	Mann-Whitney U Test	
7	A	5	UF: 0.627, DF: 0.025, DF+M: 0.176	No	0.222	Yes	Kruskal-Wallis test	Dunn's test
	B	3	Con: 0.844, $CoCl_2$: 0.554	Yes	0.144	Yes	Independent t-test	
	C	6	LC: 0.200, TA: 0.005	No	0.118	Yes	Mann-Whitney U Test	
	D	6	RCA: 0.715, LCA: 0.311	Yes	0.784	Yes	Independent t-test	
	G	3	UF: 0.571, DF: 0.173, DF+M: 0.065	Yes	0.214	Yes	One-way ANOVA	Tukey's test
	H	3-4	UF: 0.799, DF: 0.306, DF+M: 0.466	Yes	0.051	Yes	One-way ANOVA	Tukey's test
S1	B	3	TA: 0.212, LC: 0.964	Yes	0.439	Yes	Independent t-test	
	D	3	RCA: 0.682, LCA: 0.640	Yes	0.932	Yes	Independent t-test	
S2	B	3	UF: 0.118, DF: 0.976	Yes	0.248	Yes	Independent t-test	
	D	3	UF: 0.896, DF: 0.251	Yes	0.517	Yes	Independent t-test	
S3	B	3	UF: 0.822, DF: 0.656 DF+Mdivi1: 0.582	Yes	0.055	Yes	One-way ANOVA	Tukey's test
S4	NuDNA	3	UF: 0.465, DF: 0.332 DF+Mdivi1: 0.339	Yes	0.010	No	Welch's ANOVA	Games-Howell's test
	mtDNA	3	UF: 0.189, DF: 0.970 DF+Mdivi1: 0.054	Yes	0.362	Yes	One-way ANOVA	Tukey's test
S7	C	3	TA: 0.354, LC:0.102	Yes	0.039	No	Welch's t-test	
	D	3	TA: 0.665, LC: 0.890	Yes	0.490	Yes	Independent t-test	
	E	3	TA: 0.119, LC: 0.236	Yes	0.567	Yes	Independent t-test	
S8	T-eNOS	10	UF: 0.829, DF: 0.545	Yes	0.304	Yes	Independent t-test	

	PDrp1S637	8	UF: 0.653, DF: 0.782	Yes	0.243	Yes	Independent t-test	
	PDrp1S616	4	UF: 0.277, DF: 0.423	Yes	0.329	Yes	Independent t-test	
	T-Drp1	4	UF: 0.141, DF: 0.608	Yes	0.493	Yes	Independent t-test	
	Mfn2	3	UF: 0.172, DF: 0.707	Yes	0.518	Yes	Independent t-test	
	OPA1	4	UF: 0.950, DF: 0.052	Yes	0.086	Yes	Independent t-test	
	FIS1	3	UF: 0.111, DF: 0.677	Yes	0.254	Yes	Independent t-test	

Supplemental Figure S1

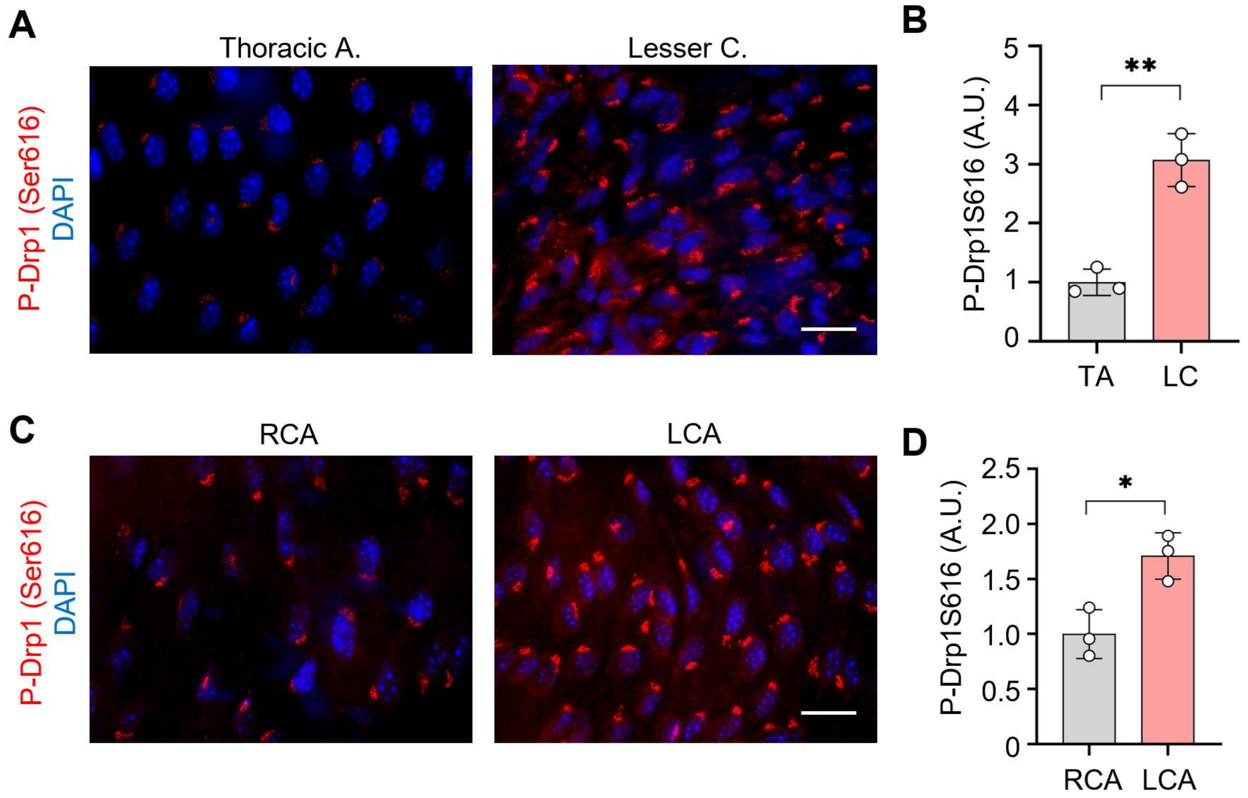

Supplemental figure S1. Phospho-Drp1 at Ser616 under UF vs. DF *in vivo*. (A) Representative micrographs of phospho Drp1 at Ser616 in ECs at TA vs. LC. Scale bar = 20 µm. (n=3). **(B)** Quantification plot of phospho Drp1 at Ser616 in ECs at TA vs. LC. **(C)** Representative fluorescence images of phospho Drp1 at Ser616 in ECs at intact RCA vs. ligated LCA. Scale bar = 20 µm. (n=3). **(D)** Quantification plot of phospho Drp1 at Ser616 in ECs at RCA vs. LCA. Data shown as means ± SD; *p<.05, **p<.01 by two-tailed independent Student's t-test; A.U. = Arbitrary unit.

Supplemental Figure S2

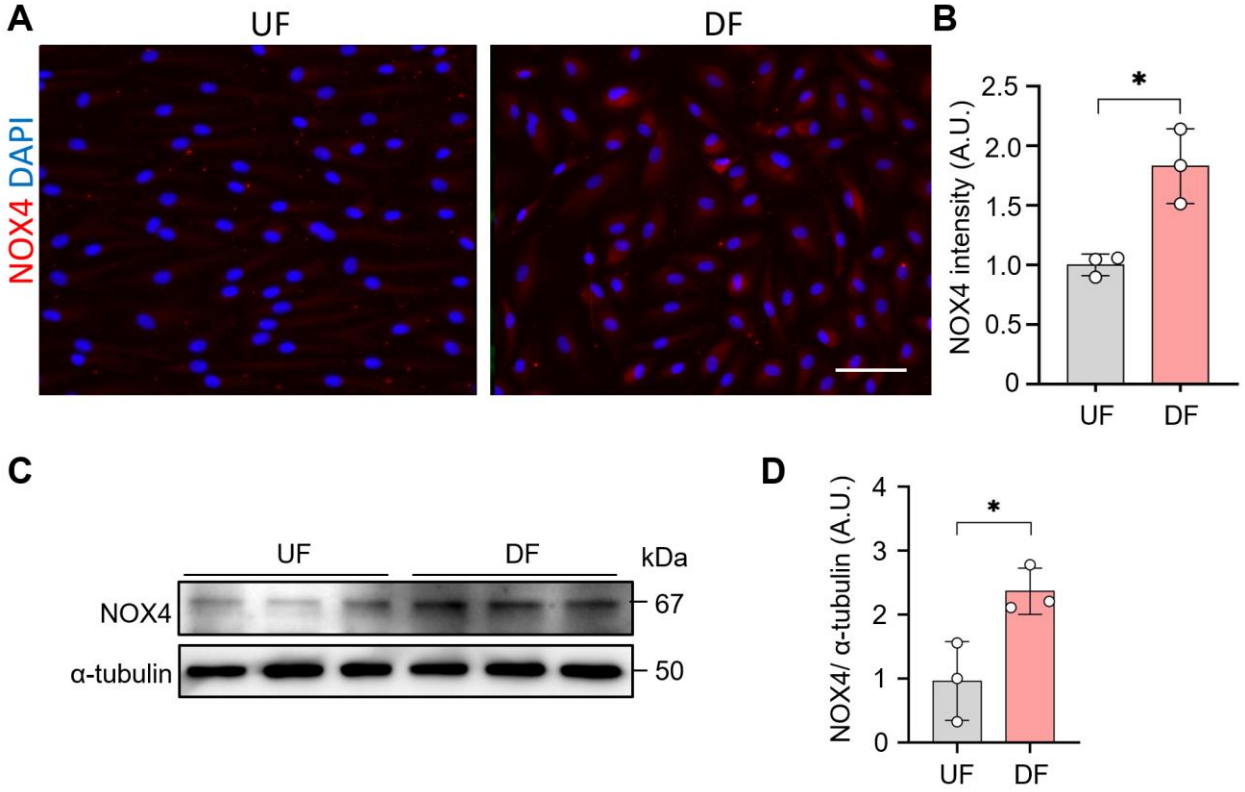

Supplemental figure S2. DF elevates NOX4 expression in HAECs. (A) Representative micrographs of NOX4 (red) and DAPI (blue) under UF (20 dyne/cm^2, 48h) vs. DF (5 dyne/cm^2, 1Hz, 48h) in HAECs. Scale bar = 100 µm. (**B)** Quantification plot of NOX4 relative intensity. **(C)** Representative immunoblot image of NOX4 under UF vs. DF in HAECs. α-tubulin was used as a loading control. **(D)** Quantification plot of NOX4 protein expression level. Data shown as means ± SD; *p<.05 by two-tailed independent Student's t-test; A.U. = Arbitrary unit.

Supplemental Figure S3

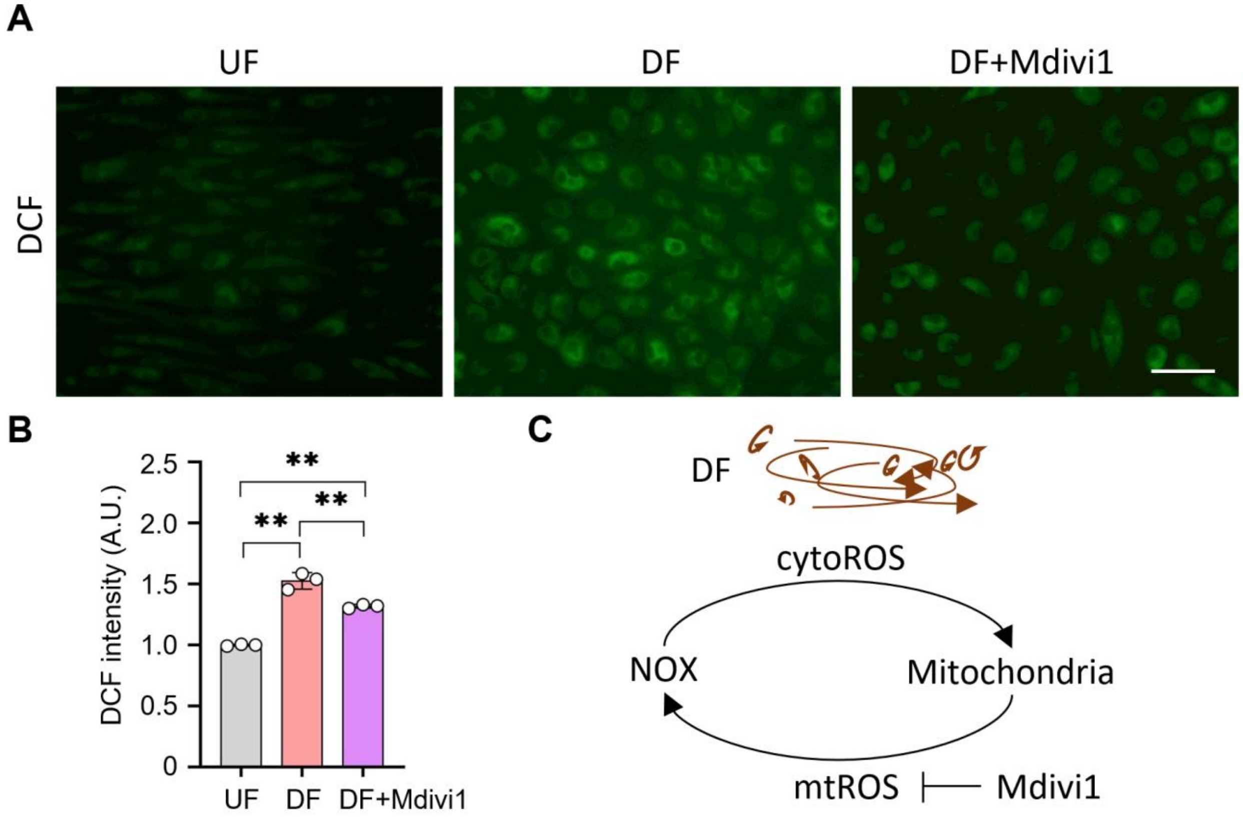

Supplemental figure S3. ROS production measured by DCF probe under UF vs. DF vs. DF+Mdivi1 in HAECs. (A) Representative micrographs of DCF fluorescence images under UF (20 dyne/cm^2, 48h) vs. DF (5 dyne/cm^2, 1Hz, 48h) vs. DF+Mdivi1 (25 µM) in HAECs. Scale bar = 100 µm. (**B)** Quantification plot of DCF relative intensity. **(C)** Disturbed flow elevates total ROS level measured by DCF, but mdivi1 treatment attenuates cellular ROS level under DF. It seems that mdivi1 treatment reduces mtROS production by inhibiting mitochondrial fragmentation, which may halt the vicious cycle under DF in HAECs. cytoROS, cytosolic ROS. mtROS, mitochondrial ROS. Data shown as means ± SD; **p<.01 by one-way ANOVA followed by Tukey's post-hoc analysis; A.U. = Arbitrary unit.

Supplemental Figure S4

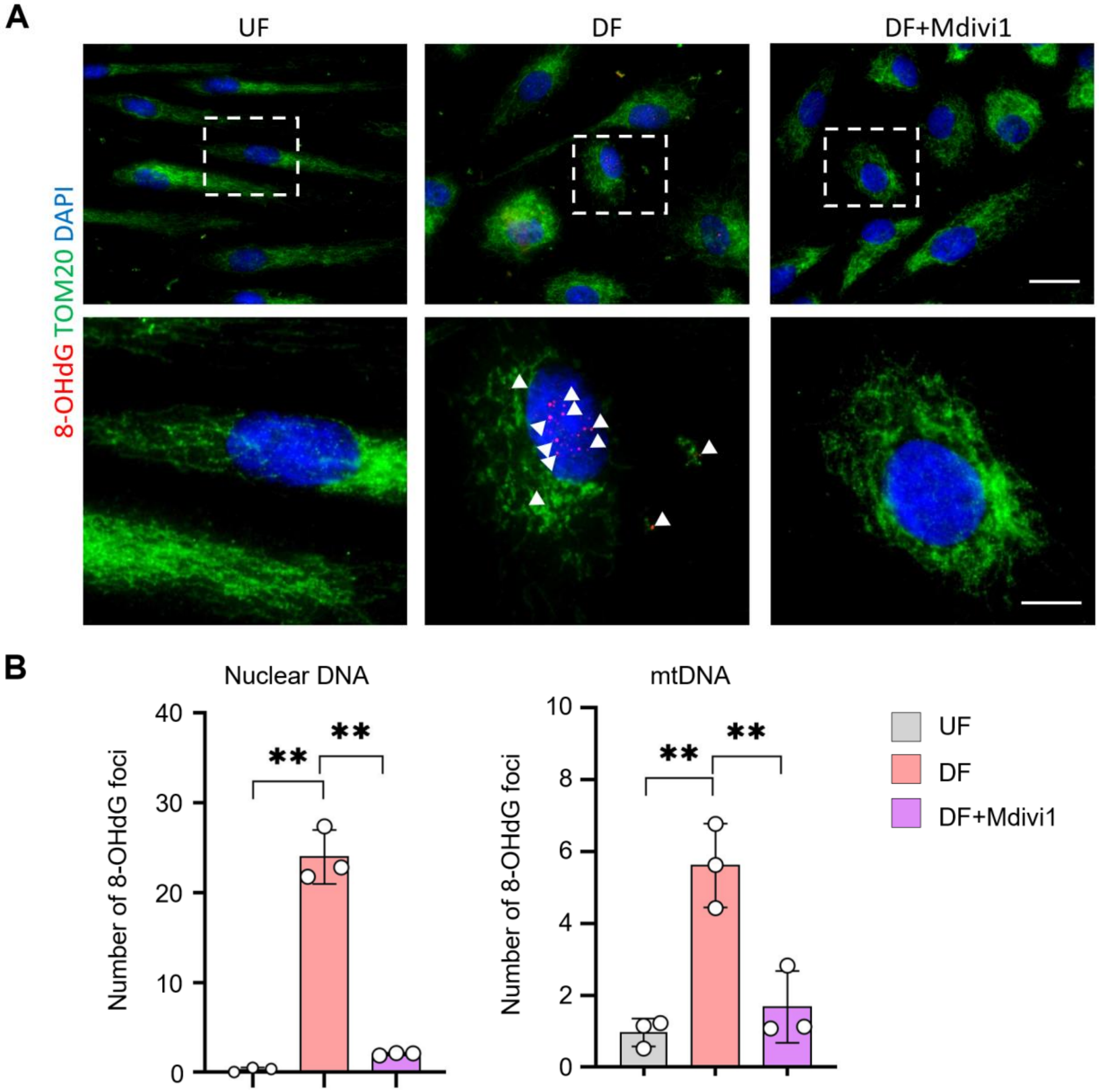

Supplemental figure S4. Oxidative damages of nuclear and mitochondrial DNA measured by 8-OHdG staining under UF vs. DF vs. DF+Mdivi1 in HAECs. (A) Representative micrographs of 8-OHdG and Tom20 (mitochondrial marker) fluorescence images under UF (20 dyne/cm^2, 48h) vs. DF (5 dyne/cm^2, 1Hz, 48h) vs. DF+Mdivi1 (25 µM) in HAECs. Scale bar = 30 µm (upper) and 10 µm (lower). (**B)** Quantification plot of nuclear and mitochondrial 8-OHdG foci number. Disturbed flow elevates both nuclear and mitochondrial oxidative damage, but mdivi1 treatment attenuates the oxidative DNA damage. It seems that mdivi1 treatment reduces mtROS production by inhibiting mitochondrial fragmentation, which may halt the vicious cycle eventually lowering nuclear and mitochondrial oxidative damages under DF in HAECs. mtDNA, mitochondrial DNA. Data shown as means ± SD; **p<.01 by Welch's ANOVA followed by Games-Howell's test (nuclear DNA) or one-way ANOVA followed by Tukey's post-hoc analysis (mtDNA); A.U. = Arbitrary unit.

Supplemental Figure S5

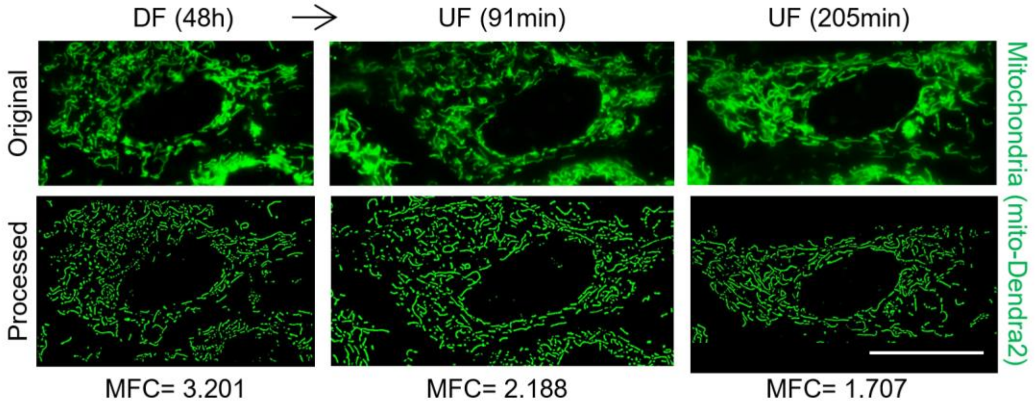

Supplemental figure S5. **Mitochondrial morphology under the transition of flow pattern from DF to UF.** Fragmented mitochondria under DF for 48 hours were gradually altered to elongated shape after the transition of the flow pattern from DF to UF. As well, this comes with a reduction of mitochondrial fission count (MFC). Green signal = mitochondria (mito-Dendra2). Scale bar = 30 µm.

Supplemental Figure S6

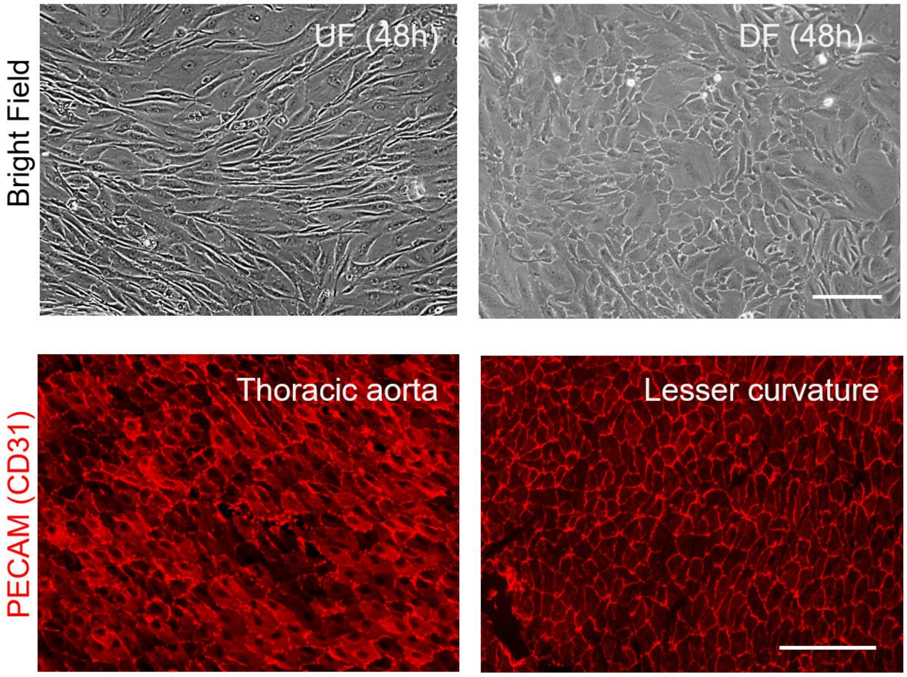

Supplemental figure S6. Comparison of endothelial cell morphology between primary mouse aortic endothelial cells under UF or DF for 48h vs. endothelium at thoracic aorta (TA) or lesser curvature. Endothelial cell (EC) morphology under 48h UF corresponds to the EC at thoracic aorta that is known to be exposed to UF, while EC morphology under DF corresponds to the EC shape at the lesser curvature of the aortic arch where DF is present. UF, unidirectional flow; DF, disturbed flow. Scale bar = 200 µm (bright field, top panel) and 100 µm (PECAM staining in red, bottom panel).

Supplemental Figure S7

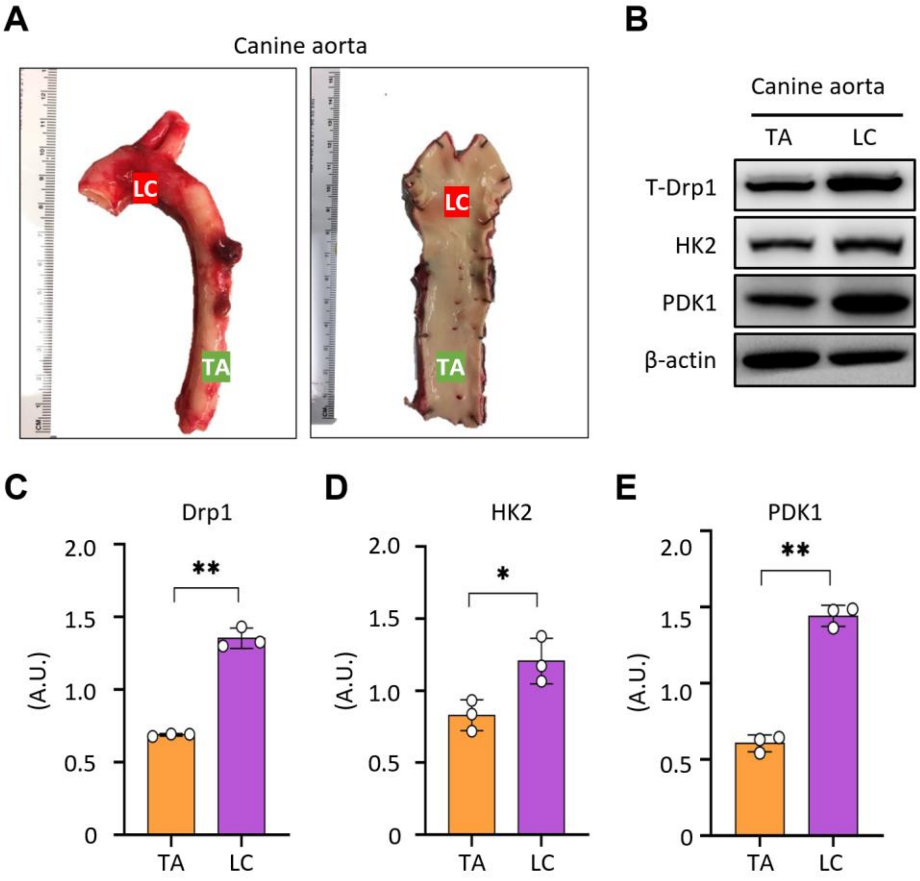

Supplemental figure S7. **DF increases mitochondrial fragmentation with elevated Drp1 activity and instigates atheroprone endothelial phenotypes *in vivo*. (A)** Representative images of aorta from canines. **(B)** Representative immunoblot images for the protein expression of T-Drp1, HK2 and PDK1 in ECs at TA vs. LC. **(C-E)** Quantification plots for immunoblot. Bar graphs are results of densitometry analyses (n=3). Data shown as means ± SD. *p<.05. **p<.01 by two-tailed independent Student's t-test (D and E) or Welch's t-test (C). A.U.=Arbitrary Unit.

Supplemental Figure S8

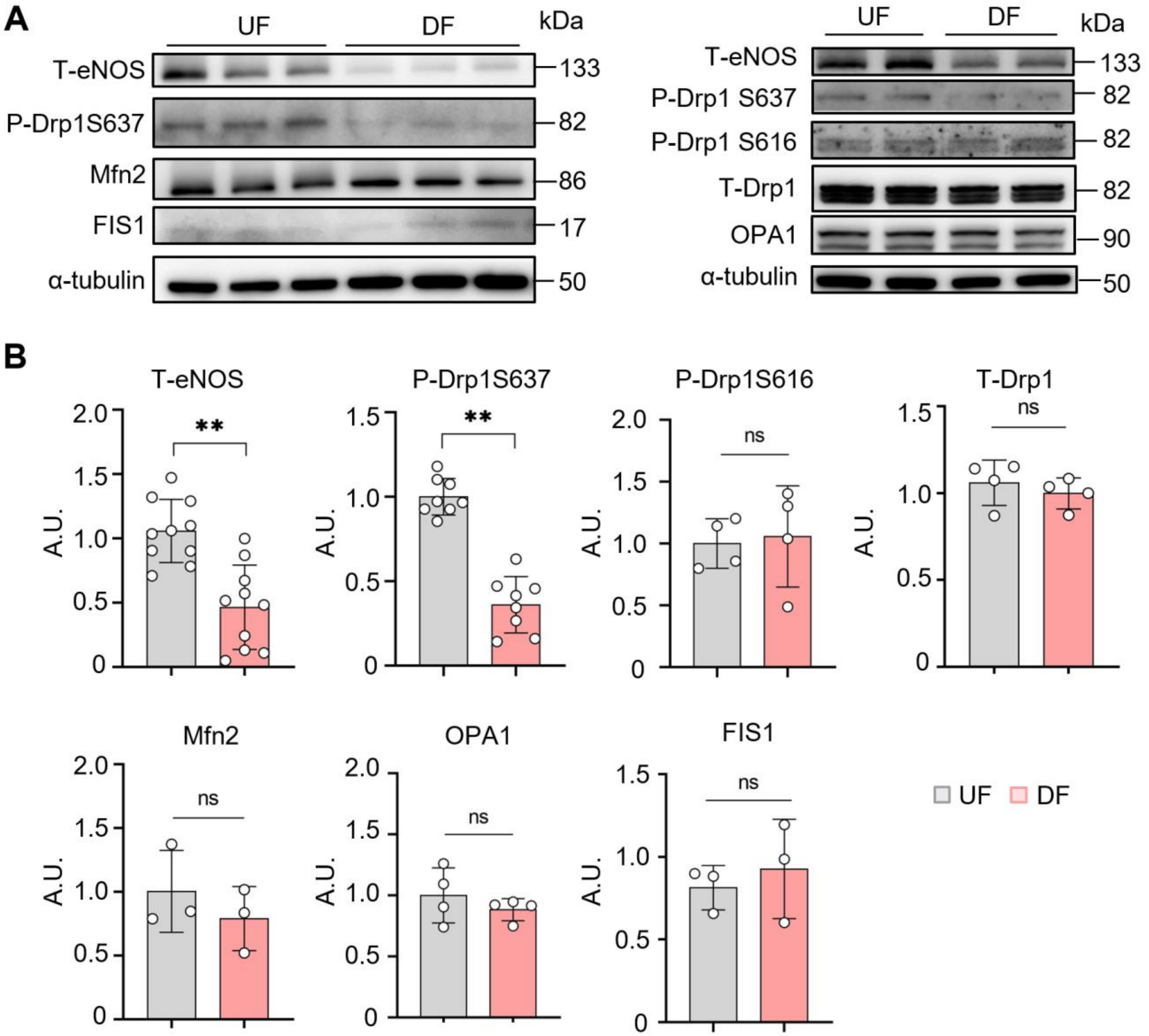

Supplemental figure S8. Mitochondria dynamics under UF vs. DF in HAECs *in vitro*. (A) Representative immunoblot images of T-eNOS, P-Drp1Ser637, P-Drp1Ser616, Mfn2, OPA1 and FIS1. α-tubulin was used as a loading control. Either UF (20 dyne/cm^2, 48h) or DF (5 dyne/cm^2, 1Hz, 48h) were applied to HAECs. **(B)** Quantification plots. Data shown as means ± SD; ns, not significant; **p<.01 by two-tailed independent Student's t-test; A.U. = Arbitrary unit.

Extended Materials and Methods

Blood vessel isolation

Mice were anesthetized with isoflurane, and the midline of the abdomen was cut and opened to expose the heart. Then, the mice were perfused with 10 ml of cold-phosphate-buffered saline (PBS) at a pressure of approximately 100 mmHg with an incision of the right atrium to release the blood followed by perfusion with a fixative (10 ml of cold 2% paraformaldehyde, PFA). For *en face* staining, different regions of the aortas and arteries including the aortic arch, carotid artery, thoracic aorta, abdominal aorta, femoral artery, and mesenteric artery, were isolated. The isolated blood vessels were post-fixed with 0.4% PFA overnight at 4℃.

En face immunostaining

PFA-fixed vessels were washed three times with PBS and incubated with 0.1M Glycine in 2% bovine serum albumin (BSA) in PBS for 30 min at room temperature (RT). Then, the vessels were permeabilized by incubating with 0.3% Triton-X in 2% BSA/PBS for 30 min at RT. The vessels then were incubated with primary antibodies in 2% BSA/PBS overnight at 4℃ with gentle agitation. Primary antibodies were from following sources: Total Drp1 (BD Biosciences, #610296), Phospho-Drp1 S637 (Cell Signaling Technology, #6319), Phospho-Drp1 S616 (Cell Signaling Technology, #4494), VCAM-1 (BD Pharmingen, #550547), HIF-1α (NOVUS, NB100-479). After rinsing in 2% BSA/PBS three times, the vessels were incubated with secondary antibodies in 2% BSA/PBS for 2 hours at RT. The vessels were placed on a slide glass and cut longitudinally and mounted in DAPI Fluoromount-G (Southern Biotech). Mitochondrial morphology was imaged under a fluorescence microscope (AxioImager, Zeiss) with the 20x and 63x oil objective lens. Endothelial-specific mito-Dendra2 signal in EC-mitochondria was excited by the 488 nm laser.

ECs were identified by co-staining with EC markers such as platelet endothelial cell adhesion molecule (PECAM-1, CD31, #MAB1398Z, Millipore) or vascular endothelial (VE)-cadherin (VE-Cadherin, CD144, #14-1441-81, Invitrogen).

Primary mouse aortic endothelial cell (MAEC) isolation

Primary MAECs were isolated from the aorta of EC-PhAM mice following the protocol described previously.[1] Briefly, mice were anesthetized with isoflurane, and the midline of the abdomen was cut and opened to expose the heart, and then the mice were perfused with 10 ml of PBS containing 1,000U/ml of heparin followed by an incision of the right atrium to release the blood. The aorta was isolated and immersed in 20% FBS-M199 containing 1,000 U/ml of heparin. Under a microscope, the connective tissues were removed rapidly, and then a 24-gauge cannula was introduced to the proximal portion of the aorta. The site where the cannula was positioned was ligated with a silk thread, and the inside of the lumen was washed with serum-free M199 media. After washing, the distal portion of the aorta was also ligated, and then the aorta was filled with a collagenase type II solution (2 mg/ml, dissolved in serum-free M199). The aorta was incubated for 45 min at 37°C, and MAECs were dissociated from the aorta by flushing with 5`ml of pre-warmed M199 containing 20% FBS. The MAECs were collected by centrifugation at 1,200 rpm for 5 min. The cells were re-suspended with 100 ul of 20% FBS-M199 and seeded on µ-slide (ibidi) that was coated with 0.1% collagen Type I in advance. After a 2-hour incubation at 37°C, the medium was changed to complete M199 medium with high ECGS (100 ug/ml).

Shear stress application

An ibidi *in vitro* pump system (ibidi, Germany) was utilized for applying two different flow conditions, either unidirectional laminar flow (UF, 20 dyne/cm^2) or disturbed flow (DF, +/- 5 dyne/cm^2, 1Hz), across the endothelial monolayer. The perfusion sets and fluidic units were kept and operated in a 37°C and 5% CO_2 incubator. When endothelial cells formed a confluent cell layer, each type of shear stress was applied to the ibidi μ-slides for 48 hours.

Live cell imaging

Live cell imaging was performed for measuring mitochondrial morphology, 2-NBDG uptake, BODIPY uptake, and MitoSOX intensity. HAECs or primary cultured MAECs from EC-PhAM mice were seeded into ibidi μ-slides and exposed to either UF or DF for 48 hours using the ibidi pump system. Temperature and CO_2 were maintained at 37°C and 5% CO_2 by a stage-top incubation system (ibidi, Germany). Images were acquired using an epifluorescence inverted microscope (ZEISS AxioVert.A1) with a 20x or 63x objective oil lens.

Glucose uptake measurement using 2-NBDG

HAECs were seeded in μ-slides (ibidi) and subjected to either UF or DF for 48 hours. Immediate after the flow applications, cells were subjected to glucose uptake assay using 2-NBDG glucose uptake assay kit (#K682-50, BioVision). Briefly, cells were incubated with 2-NBDG glucose uptake mix at 37°C for 30 min. After the incubation, the cells were washed three times with pre-warmed PBS, and then analysis buffer provided was added for live cell imaging. 488nm excitation laser was used to detect 2-NBDG fluorescent signal. ImageJ (NIH) was used for the quantification of fluorescent intensity.

Fatty acid uptake measurement using BODIPY probe

HAECs were seeded in μ-slides (ibidi), and either UF or DF was applied to ECs for 48 hours. Immediately after the flow applications, cells were incubated with BODIPY (5uM, C1-BODIPY 500/510 C12, D3823, Molecular probes) fatty acid probe at 37°C for 30 min. After the incubation, the cells were washed three times with pre-warmed PBS, and fatty uptake was visualized under an inverted fluorescent microscope with 532nm excitation laser. ImageJ (NIH) was used for the quantification of fluorescent intensity.

Canine aorta and EC protein sample

The freshly isolated canine aortas were obtained (One-year-old male dogs; n=3). The aortas were rapidly dissected and immersed in ice-cold PBS. The aortas were cut open and washed quickly with cold PBS. A plastic mold was placed on the surface of the vessel (EC side), and 200 µl of cold-PBS was added. The EC surface was scraped by a scraper, and the cold-PBS containing ECs were collected. The collected cells in PBS were centrifuged at 1,500 rpm for 5 min at 4ºC. The supernatant was discarded, and 100 ul RIPA lysis buffer (including protease inhibitor and phosphatase inhibitor) was added to the cell pellet. The lysed samples were frozen at -80ºC until use.

Mitochondrial morphology quantification

A quantitative analysis of mitochondrial morphology was performed based on the methods described previously.[2] Briefly, obtained images were processed using ImageJ (NIH) to subtract backgrounds and subjected to kernel convolution (matrix h described below) to emphasize the

edges of each mitochondrial particle. Then, the processed images were subjected to binary conversion.

$$h = \begin{bmatrix} 0 & 0 & -1 & -1 & -1 & 0 & 0 \\ 0 & -1 & -1 & -1 & -1 & -1 & 0 \\ -1 & -1 & +3 & +3 & +3 & -1 & -1 \\ -1 & -1 & +3 & +4 & +3 & -1 & -1 \\ -1 & -1 & +3 & +3 & +3 & -1 & -1 \\ 0 & -1 & -1 & -1 & -1 & -1 & 0 \\ 0 & 0 & -1 & -1 & -1 & 0 & 0 \end{bmatrix}$$ (Koopman et al., 2005 [3])

After the binary conversion, individual mitochondrial particles were analyzed using the "analyze particles" function of ImageJ for calculating circularity and major/minor axes. Form factor (FF: the reciprocal of circularity value/(perimeter^2/4π*area)) and aspect ratio (AR: major axis/minor axis of an ellipse equivalent to the object) were calculated, and then a scatter plot of AR versus FF was generated for each image. AR, a measure of mitochondrial length, and FF, a measure of both mitochondrial length and branching, have a minimum value of 1 when it is a perfect, small circle, and the value increases as mitochondria become elongated and branched. Furthermore, the mitochondrial morphology of each cell was categorized into either 1. fragmented, 2. tubular, or 3. elongated (Supplemental figure 1). Then, the number of cells that are classified into each category were counted and displayed as a percentage of the total cell number. Over 200 cells were analyzed for each condition. In addition, mitochondrial fragmentation counts (MFC) were calculated based on the method previously described.[4] The mitochondrial fragmentation count (MFC) quantifies discrete mitochondrial particles, and greater fission results in higher MFC values. Using the binary images, mitochondrial segments were identified and counted with analyze particles function of ImageJ, and the number was normalized to the total mitochondrial area to obtain the MFC for each imaged cell (MFC = mitochondria number / total mitochondrial area).

Immunoblotting

Immunoblotting was performed as described previously.[5] Briefly, cells were washed three times with cold PBS, and RIPA buffer (10 mM Tris-HCl, 5 mM EDTA, 150 mM NaCl, 1% Triton X-100, 0.1% SDS, 1% Deoxycholate, pH 7.5) was added to lyse the cells. Collected RIPA samples were centrifuged at 16,000g for 15 min at 4°C, and the supernatants were collected and subjected to a BCA protein assay (Pierce™ BCA Protein Assay Kit, #23225) to quantify the protein concentration. The resulting protein samples were subjected to SDS-PAGE and transferred to a polyvinylidene difluoride membrane. Subsequently, the membrane was blocked with 5% nonfat dry milk in Tris-buffered saline-Tween 20 (TBST) for 20 min at RT and incubated overnight with respective primary antibodies. The membranes were then washed three times in TBST and incubated with HRP-conjugated secondary antibodies for an hour. Then, the membranes were washed three times with TBST, and membranes were subjected to standard enhanced chemiluminescence (Thermo Fisher Scientific) method for visualization. Antibodies were from following sources: Drp1 (BD Biosciences, #610296), CD144 (Invitrogen, #14-1441-81), P-Drp1 Ser637 (Cell Signaling Technology, #6319), β-actin (Sigma-Aldrich, A1978), VCAM-1 (Santa Cruz, #sc-13160), CD31 (Millipore, #MAB1398Z), PDK1 (Santa Cruz, #sc-515944), HK2 (Santa cruz, #sc-374091), T-eNOS (BD Biosciences, #610296), OPA1 (BD Biosciences, #612606), Mfn2 (Santa Cruz, sc-100560), Fis1 (Sigma-Aldrich, HPA017430), NOX4 (NOVUS, #NB110-58849), anti-HIF-1α (NOVUS, NB100-479) and α-tubulin (Sigma-Aldrich, #T9026).

Immunostaining

Cells were fixed with 4% PFA in PBS for 15 min at RT and followed by washing with PBS three times. The fixed cells were subjected to blocking with staining buffer (10% normal goat serum in PBS containing 0.3% Triton X-100) for 1 hr at RT, and then cells were incubated with primary

antibody diluted in staining buffer for at 4℃ overnight. The cells were washed with PBS three times and subsequently incubated with secondary antibody for 2 hrs at RT. After washing with PBS three times, cells were mounted with DAPI fluoromount-G (SourternBiotech, 0100-20), and images were acquired using a fluorescence microscope (AxioImager, Zeiss). Antibodies were from following sources: Anti-CD31 (Millipore, MAB1398Z), anti-HIF-1α (NOVUS, NB100-479), anti-VCAM-1 (Santa Cruz, sc-13160), and NOX4 (NOVUS, #NB110-58849) were used for immunostaining.

Transmission electron microscopy (TEM)

HUVECs were seeded and grown in μ-slides (ibidi, Germany) and transfected with either scramble control siRNA (Invitrogen, #462001) or siDrp1 (Invitrogen, #HSS115288) using Lipofectamine RNAiMAX transfection reagent (Thermo Fisher, #13778100). The HUVECs were subjected to either UF (20 dyne/cm^2) or DF (5 dyne/cm^2, 1Hz) for 48h using ibidi pump system. The HUVECs were washed with PBS and fixed with 2.5% glutaraldehyde, 2.0% paraformaldehyde in 0.1M sodium cacodylate buffer, pH7.4, overnight at 4℃. After subsequent buffer washes, the samples were post-fixed in 2.0% osmium tetroxide for 1 hour at room temperature, and then washed again in buffer followed by dH_2O. After dehydration through a graded ethanol series, the tissue was infiltrated and embedded in EMbed-812 (Electron Microscopy Sciences, Fort Washington, PA). Thin sections were stained with uranyl acetate and lead citrate and examined with a JEOL 1010 electron microscope fitted with a Hamamatsu digital camera and AMT Advantage image capture software.

Oxidative stress measurement

Cellular oxidative stress were measured using CM-H_2DCFDA General Oxidative Stress Indicator (Molecular Probes, C6827) following manufacturer's instructions. HAECs were seeded and grown in μ-slides (ibidi, Germany), and 10 μM CM-H_2DCFDA were incubated with HAECs for 20 min (37℃). The HAECs were subjected to either UF (20 dyne/cm^2) or DF (5 dyne/cm^2, 1Hz) or DF (5 dyne/cm^2, 1Hz) with mdivi-1 (25 μM, Sigma-Aldrich, M0199) for 48h using ibidi pump system, and DCF intensity was measured under an inverted fluorescence microscope (Zeiss, Observer.Z1).

Oxidative nuclear and mtDNA damage quantification by 8-OHdG staining

Quantification of oxidative nuclear and mtDNA damage by 8-OHdG staining were documented previously.[1-3] HUVECs were subjected to either UF (20 dyne/cm^2) or DF (5 dyne/cm^2, 1Hz) or DF (5 dyne/cm^2, 1Hz) with mdivi-1 (25 μM, Sigma-Aldrich, M0199) for 48h using ibidi pump system. Cells were fixed with 4% PFA in PBS for 15 min at RT and followed by washing with PBS three times. The fixed cells were subjected to blocking with staining buffer (10% normal goat serum in PBS containing 0.3% Triton X-100) for 1 hour at RT, and then cells were incubated with 8-OHdG (E-8) antibody (Santa Cruz, sc-393871) and Tom20 antibody (Cell Signaling Technology, D8T4N, 42406) diluted in staining buffer for at 4℃ overnight. The cells were washed with PBS three times and subsequently incubated with secondary antibodies for 2 hours at RT. After washing with PBS three times, cells were mounted with DAPI fluoromount-G (SourternBiotech, 0100-20), and images were acquired using a fluorescence microscope (Axioimager, Zeiss). 8-OhdG signals that are localized with DAPI were considered as nuclear oxidative damage, and those signals that are localized with mitochondria (Tom20) were considered as mtDNA oxidative damage. Average number of 8-OHdG foci in nucleus and mitochondria per cell was quantified.

Plasmid DNA transfection

mCherry-Drp1 was a gift from Gia Voeltz (Addgene plasmid # 49152 ; http://n2t.net/addgene:49152 ; RRID:Addgene_49152). The plasmid DNA was purified using the QIAGEN Plasmid Mini Kit (QIAGEN, #12123). Transfections of mCherry-Drp1 were performed using CytofectTM Endothelial Cell Transfection Kit (Cell Applications, INC, #TF101K) according to the manufacturer's recommendation.

References

1. Kobayashi M, Inoue K, Warabi E, Minami T and Kodama T. A simple method of isolating mouse aortic endothelial cells. *Journal of atherosclerosis and thrombosis*. 2005;12:138-42.
2. Kim B, Kim JS, Yoon Y, Santiago MC, Brown MD and Park JY. Inhibition of Drp1-dependent mitochondrial division impairs myogenic differentiation. *American journal of physiology Regulatory, integrative and comparative physiology*. 2013;305:R927-38.
3. Koopman WJ, Verkaart S, Visch HJ, van der Westhuizen FH, Murphy MP, van den Heuvel LW, Smeitink JA and Willems PH. Inhibition of complex I of the electron transport chain causes O2-. -mediated mitochondrial outgrowth. *American journal of physiology Cell physiology*. 2005;288:C1440-50.
4. Hong Z, Kutty S, Toth PT, Marsboom G, Hammel JM, Chamberlain C, Ryan JJ, Zhang HJ, Sharp WW, Morrow E, Trivedi K, Weir EK and Archer SL. Role of dynamin-related protein 1 (Drp1)-mediated mitochondrial fission in oxygen sensing and constriction of the ductus arteriosus. *Circulation research*. 2013;112:802-15.
5. Kim B, Lee H, Kawata K and Park JY. Exercise-mediated wall shear stress increases mitochondrial biogenesis in vascular endothelium. *PloS one*. 2014;9:e111409.
